# SNX27:Retromer:ESCPE-1-mediated early endosomal tubulation impacts cytomegalovirus replication

**DOI:** 10.3389/fcimb.2024.1399761

**Published:** 2024-09-18

**Authors:** Igor Štimac, Marina Marcelić, Barbara Radić, Ivona Viduka, Gordana Blagojević Zagorac, Silvija Lukanović Jurić, Carmen Rožmanić, Martin Messerle, Ilija Brizić, Pero Lučin, Hana Mahmutefendić Lučin

**Affiliations:** ^1^ Department of Physiology, Immunology and Pathophysiology, Faculty of Medicine, University of Rijeka, Rijeka, Croatia; ^2^ University North, University Center Varaždin, Varaždin, Croatia; ^3^ Center for Proteomics, Faculty of Medicine, University of Rijeka, Rijeka, Croatia; ^4^ Institute of Virology, Hannover Medical School, Hannover, Germany

**Keywords:** Cytomegalovirus, assembly compartment, beta-herpesvirus secondary envelopment, sorting nexin 27, tubular endosomes, retromer, ESCPE-1

## Abstract

**Introduction:**

Cytomegaloviruses (CMVs) extensively reorganize the membrane system of the cell and establish a new structure as large as the cell nucleus called the assembly compartment (AC). Our previous studies on murine CMV (MCMV)-infected fibroblasts indicated that the inner part of the AC contains rearranged early endosomes, recycling endosomes, endosomal recycling compartments and trans-Golgi membrane structures that are extensively tubulated, including the expansion and retention of tubular Rab10 elements. An essential process that initiates Rab10-associated tubulation is cargo sorting and retrieval mediated by SNX27, Retromer, and ESCPE-1 (endosomal SNX-BAR sorting complex for promoting exit 1) complexes.

**Objective:**

The aim of this study was to investigate the role of SNX27:Retromer:ESCPE-1 complexes in the biogenesis of pre-AC in MCMV-infected cells and subsequently their role in secondary envelopment and release of infectious virions.

**Results:**

Here we show that SNX27:Retromer:ESCPE1-mediated tubulation is essential for the establishment of a Rab10-decorated subset of membranes within the pre-AC, a function that requires an intact F3 subdomain of the SNX27 FERM domain. Suppression of SNX27-mediated functions resulted in an almost tenfold decrease in the release of infectious virions. However, these effects cannot be directly linked to the contribution of SNX27:Retromer:ESCPE-1-dependent tubulation to the secondary envelopment, as suppression of these components, including the F3-FERM domain, led to a decrease in MCMV protein expression and inhibited the progression of the replication cycle.

**Conclusion:**

This study demonstrates a novel and important function of membrane tubulation within the pre-AC associated with the control of viral protein expression.

## 1 Introduction

Cytomegaloviruses (CMVs), large DNA viruses that belong to the beta-herpesvirus subfamily, cause asymptomatic infections in about 70% of the population, which can be associated with many pathophysiological conditions and can cause severe forms in immunocompromised hosts (Gugliesi et al., 2020). CMV infection leads to an extensive restructuring of the cell, the establishment of multiple membraneless compartments (Sanchez and Britt, 2022; Mahmutefendić Lučin et al., 2023), and a complete reorganization of the cell membrane system (reviewed by Wofford et al., 2020; Mosher et al., 2022; Turner and Mathias, 2022). These changes are associated with a reorganization of cell physiology, and their understanding is essential for understanding CMV pathogenesis and the further development of host-directed antiviral therapy (Kumar et al., 2020). Most studies on CMV biology are conducted on human CMV (HCMV) and murine CMV (MCMV). These viruses are relatively closely related, and many aspects of their biology appear to be similar (Fisher and Lloyd, 2021).

The reorganization of the membrane system gives rise to a new order of membrane organelles, including the development of the assembly compartment (AC) (Wofford et al., 2020; Mosher et al., 2022). The AC is a structure, as large as the nucleus, that contains relocated membrane organelles arranged in an order that is different from uninfected cell. The AC comprises displaced and expanded Golgi stacks in a ring-like organization called the outer AC, surrounding expanded elements of the early endosomal (EE) system, recycling endosomes (REs), and the trans-Golgi network (TGN), called the inner AC (Das et al., 2007; Das and Pellett, 2011; Lučin et al., 2020). The AC membrane elements displace the endoplasmic reticulum (ER) and late endosomes (LEs) to the periphery of the cell (Beltran et al., 2016; Lučin et al., 2020). The development of the AC is initiated in the early (E) phase of infection by a series of membrane reorganization events that establish the basic configuration (Karleuša et al., 2018; Lučin et al., 2018, 2020; Štimac et al., 2021), referred to as pre-AC, which further matures in the late (L) phase of infection after viral DNA replication and expression of late proteins, including most of the structural proteins required for virion assembly. Structural proteins that assemble into capsids are transported into the nucleus, while most of the proteins responsible for the assembly of the tegument and virion envelope are concentrated in the AC (Close et al., 2018). Capsids assembled in the nucleus pass the nuclear membrane, are released into the cytosol, and finally embedded in condensates of tegument proteins. The tegumented capsids further acquire a membrane envelope in a process known as secondary envelopment (Close et al., 2018). Since most envelopment events occur at the membranes within the AC (Schauflinger et al., 2013), an important function of the AC is the final envelopment of virions and their packaging into membrane vehicles suitable for the release of virions from the cell. However, it is reasonable to expect that such a major reorganization of the cell’s membrane physiology may have additional, important consequences for the viral life cycle.

The site and mechanism of the final envelopment are not known. Existing data suggest envelopment at membranes derived from EEs (Radsak et al., 1996; Fraile-Ramos et al., 2002), the RE system (Krzyzaniak et al., 2009; Mahmutefendić Lučin et al., 2022), and the TGN (Jarvis et al., 2002; Homman-Loudiyi et al., 2003; Cepeda et al., 2010) by a mechanism that may involve budding of the nucleocapsid into large organelles or wrapping of membranes around tegumented virions. Electron microscopy studies (Maninger et al., 2011; Schauflinger et al., 2013; Taisne et al., 2019; Momtaz et al., 2021; Flomm et al., 2022; Wedemann et al., 2022) provide evidence for both. Wrapping-based envelopment is of particular importance for MCMV, as it generates multicapsid virions (Maninger et al., 2011).

Regardless of the mechanism, the envelopment process requires a membrane composition capable of concentrating virion envelope proteins, accommodating large biomolecular condensates of tegument components, and growing capacity to wrap around large structures such as tegumented virions, particularly multicapsid virions in MCMV-infected cells (Lučin et al., 2023). Recently described wrapping of membranes derived from REs in the biogenesis of autophagosomes (Puri et al., 2023) may be a clue in the identification of a mechanism utilized for wrapping of tegumented virions. Thus, among the spatiotemporal ordering of complex machinery to establish such a membrane composition, the study of cargo retrieval mechanisms and mechanisms associated with membrane tubulation within the AC may be essential for understanding the biogenesis of the AC and subsequently the identification of the envelopment egress mechanism of beta-herpesviruses. Both cargo retrieval and wrapping-based envelopment are associated with tubulation within the endosomal and TGN systems and the mechanisms associated with endosomal recycling (Lučin et al., 2023).

Tubulation is a property of membranes within the inner AC that starts very early in infection (Lučin et al., 2020; Wofford et al., 2020; Mosher et al., 2022). Traces of host cell components within the virions identified by proteomic analysis indicate that membranes belonging to the endosomal recycling system may be used for envelopment (Turner et al., 2020; Mahmutefendić Lučin et al., 2022). Thus, cargo sorting into the recycling domain and subsequent tubulation relies mainly on sequence-dependent retrieval complexes, which include either adaptor protein (AP) complexes associated with the activation of Arf GTPases at the membranes (D’Souza et al., 2014) or a heterotrimer consisting of vacuolar protein sorting 35 (Vps35), Vps26 and Vps29, known as the Retromer complex (Cullen and Steinberg, 2018), or a heterotrimer consisting of DSCR3, C16orf62, and Vps29, known as the Retriever complex (McNally and Cullen, 2018). For cargo sorting and retrieval, Retromer and Retriever are additionally complexed with one or more sorting nexins (SNXs), cellular proteins characterized by a PX domain (phox homology) that dominantly recognizes phosphatidylinositol-3-phosphate (PI3P) at the cytosolic leaflet of endosomal compartments (Gallon and Cullen, 2015; Cullen and Steinberg, 2018). Some of the best understood are SNX3 and SNX27, which bind Retromer, and SNX17, which binds Retriever (Cullen and Korswagen, 2012). They form distinct subdomains on EEs and provide a platform for sorting cargo from the degradation pathway towards different recycling pathways to the plasma membrane (PM) or the TGN (Cullen and Steinberg, 2018). SNX27 has three major domains: an N-terminal PDZ domain (PSD95, disks large and zona occludens), a central PX domain, and a C-terminal FERM domain (4.1/ezrin/radixin/moesin) (Steinberg et al., 2013; Chandra et al., 2021; Simonetti et al., 2022). The PDZ domain sorts out cargo proteins from the lysosomal pathway by recognizing the type I PDZ binding motif in numerous recycling proteins and recruits Retromer by binding the Vps26 Retromer component. The PX domain recognizes the PI3P-rich phospholipid composition, while the FERM domain recruits the SNX1/SNX2:SNX5/SNX6 complex, known as the ESCPE-1 complex (endosomal SNX-BAR sorting complex for promoting exit 1), which initiates and promotes tubulation (Yong et al., 2021; Simonetti et al., 2022). Therefore, the SNX27:Retromer:ESCPE-1 complexes play an important role in sorting recycling cargo and promoting tubulation, two functions that can be utilized within the pre-AC and AC of CMV-infected cells.

The aim of this study was to investigate the role of SNX27:Retromer:ESCPE-1 complexes in the biogenesis of pre-AC in MCMV-infected cells and, subsequently, their role in the secondary envelopment and release of infectious virions. Here we show that SNX27:Retromer:ESCPE1-mediated tubulation is essential for the establishment of a Rab10-decorated subset of membranes within the pre-AC, a function that requires an intact F3 subdomain of the SNX27 FERM domain. Suppression of SNX27-mediated functions resulted in an almost tenfold decrease in the release of infectious virions. However, these effects cannot be directly linked to the contribution of SNX27:Retromer:ESCPE-1-dependent tubulation to envelopment, as suppression of these components, including the F3-FERM domain, led to a decrease in MCMV protein expression and inhibited progression through the replication cycle. Thus, this study reveals a novel and important function of membrane tubulation within the pre-AC associated with the control of viral protein expression.

## 2 Materials and methods

### 2.1 Cell lines, and cell culture

The murine fibroblast-like cell lines Balb3T3 (American Type Culture Collection, clone A31, ATCC CCL-163, Manassas, VA, USA) and NIH3T3 (ATCC CRL-163) were used for the experiments and primary murine embryonic fibroblasts (MEFs) from 17-day-old BALB/c mouse embryos were used for virus production and plaque assay. The cells were cultured in 10-cm dishes for propagation and divided into appropriate plates for the experiments once they were 80-90% confluent. For cell culture at 37°C and 5% CO_2_, Dulbecco’s Modified Eagle’s Medium (DMEM) supplemented with 10% (5% for MEF) fetal bovine serum (FBS), 2 mM L-glutamine, 100 mg/ml streptomycin and 100 U/ml penicillin (all reagents from Gibco/Invitrogen, Grand Island, NY, USA) was used.

### 2.2 Viruses and infection conditions

The recombinant virus Δm138-MCMV (ΔMC95.15) with the deletion of fcr1 (m138) gene (Crnković-Mertens et al., 1998) was regularly used in the experiments to avoid FcR-mediated non-specific binding of antibody reagents. Wild-type (wt) MCMV (strain Smith, ATCC VR-194) was used to calibrate the plaque assay. To monitor MCMV replication by flow cytometry, we used C3X-GFP MCMV (MCMV-GFP), a recombinant virus expressing green fluorescent protein (GFP) in the early phase of infection (Angulo et al., 2000). MCMV stocks were produced, and cells were infected according to standard procedures (Brizić et al., 2018). Cells were infected with 1 PFU/cell at a multiplicity of infection (MOI) of 10 after increasing infectivity by centrifugation. The efficiency of infection was monitored by intracellular detection of Immediate-Early-1 protein (pIE1) as previously described (Lučin et al., 2020).

### 2.3 Antibodies and reagents

Antibodies against proteins regulating endocytic transport were monoclonal (mAb) or polyclonal (pAb) as follows: rabbit monoclonal IgG anti Rab10 (Cell Signaling Inc., Danvers, MA, USA; Cat. No. 8127), rabbit pAbs against SNX27 (Abcam, Cambridge, UK; Cat. No. ab241128) and GRASP65 (Thermo Fisher Scientific, Waltham, MA, USA; Cat. No. PA3-910), mouse mAbs from Santa Cruz Biotechnology (Dallas, USA) against SNX1 (IgG_1_, Cat. No. sc-376376), SNX2 (IgG_2b_, Cat. No. sc-390510), SNX27 (IgG_2b_, Cat. No. sc-515707) and Vps35 (IgG_2b_, Cat. No. sc-374372), and mouse mAbs against actin (IgG_1_, Millipore, Burlington, Massachusetts, USA; Cat. No. MAB150).

Monoclonal antibodies against MCMV proteins were produced, purified, and verified by the University of Rijeka Center for Proteomics, (https://products.capri.com.hr/shop/?swoof=1&pa_reactivity=murine-cytomegalovirus; accessed on February 11, 2024). These antibodies included: mouse mAbs IgG_1_ (clone CROMA101) and IgG_2a_ (clone IE1.01) against pm123/pIE1, mAb IgG_1_ (clone CROMA103) against pM112-113 (E1), mAb IgG_1_ (clone CROMA 229) against pm06, mAb IgG_1_ (clone M55.01 for Western blot) against pM55/gB, mouse monoclonal anti M57 (clone M57.02), mAb IgG_1_ against pM74 (clone 74.01), and mAb IgG_1_ (clone M116.02) against pM116.

Alexa Fluor (AF)^488^-, AF^555^-, AF^594^-conjugated (Molecular Probes; Leiden, The Netherlands) and AF^680^-conjugated (Jacksons Laboratory, Bar Harbor, ME, USA) antibodies against mouse IgG_1_, mouse IgG_2a_, mouse IgG_2b_ and rabbit Ig were used as secondary antibodies for immunofluorescence analysis. Goat anti-rabbit and goat anti-mouse antibodies conjugated to HRP were used for Western blot analysis (Jackson Laboratories, Bar Harbor, ME, USA). DAPI (4,6-diamidino-2-phenylindole dihydrochloride) was from Thermo Fisher Scientific (Waltham, MA, USA; Cat. No. D1306). Puromycin was obtained from Santa Cruz Biotechology Inc., Dallas, USA. Propidium iodide and other chemicals were obtained from Sigma-Aldrich Chemie GmbH (Schnelldorf, Germany).

### 2.4 Immunofluorescence and confocal microscopy

The cells were cultured on coverslips in 24-well plates to 60-70% confluency for the experiment. Fixation was performed in 4% paraformaldehyde (20 min at r.t.) and permeabilization in 0.5% Tween 20 (20 min at 37°C). After incubation with appropriate primary antibodies and AF-conjugated secondary antibodies (60 min at 37°C), samples were embedded in Mowiol (Fluka Chemicals, Selzee, Germany)-DABCO (Sigma Chemical Co, Steinheim, Germany) in PBS containing 50% glycerol and analyzed by confocal microscopy (Leica DMI8 inverted confocal microscope (confocal part: TCS SP8; Leica Microsystems GmbH, Wetzlar, Germany) and HC PLAPO CS2 objective (63×1.40 oil). Lasers were used as follows: UV (Diode 405) for DAPI, Ar 488 for AF^488^, DPSS 561 for AF^555^ and AF^595^, and He/Ne 633 for AF^680^. The microscope was also equipped with 4 detectors, two of which are PMT and two are HyD. Images were acquired in sequential mode (515x515 pixels, z-series of 0.5 μm) using LAS (Leica Application Suite) X version 3.5.6.21594 software (Leica Microsystems GmbH, Wetzlar, Germany). The zoom factors were as follows: 0.75× (pixel size 481.47 × 481.47 nm), 1.5× (pixel size 240.74 × 240.74 nm), 3× (pixel size 120.37 × 120.37 nm), and 6× (pixel size 60.18 × 60.18 nm). The offset was set to 0-1.5% depending on the background. All samples that were compared within an experiment were imaged with the same parameters.

### 2.5 Image analysis

The presence of AC in infected cells was defined as a concentrated fluorescent signal within the angle of α ≤ 90° (Štimac et al., 2021). The ratio of AC-positive cells per one microscopic sample was counted in at least 10 fields of view. The direct counting was performed on an epifluorescence Olympus BX51 microscope equipped with a DP71CCD camera (Olympus, Tokyo, Japan) with UPlanFL N 40×/0.75 objective.

For the colocalization analysis, we calculated the Manders’ overlap coefficients (M1 and M2) of the entire z-stack (8-12 confocal sections) of images (120.37 × 120.57 nm pixel size) using the JACoP plugin (https://imagej.net/ij/plugins/track/jacop2.html, accessed on February 11, 2024) (Bolte and Cordelières, 2006). Briefly, red, green, and blue channels were split and colocalization of pixels between two selected channels was determined after background subtraction, as described previously (Marcelić et al., 2022). At least 15-20 cells were analyzed in each experiment.

### 2.6 siRNA silencing

Small interfering (si)RNA sequences were purchased as follows: non-targeting negative siRNA (1022076) and Mm_Snx27_7 sequence (SI04939543) were from Qiagen (Hilden, Germany); siRNA for SNX1 (sc-41346), siRNA for SNX2 (sc-41350) and siRNA for Vps35 (sc-63219) were from Santa Cruz Biotechnology Inc. (Dallas, USA). Cells were transfected according to the manufacturer’s guidelines: siRNA and RNAiMAX Lipofectamine Reagent (Invitrogen, Carlsbad, CA, USA) were mixed and added dropwise to the cells. The final concentration of siRNA for SNX1 was 20 nM, for SNX2 80 nM, for SNX27 60 nM and for Vps35 80 nM. After 48 hours, the cells were analyzed or used for infection.

### 2.7 Flow cytometry and detection of cells infected with C3X-GFP-MCMV

Balb3T3 cells, treated with the corresponding siRNA for 48 h, were infected with C3X-GFP-MCMV (MOI of 10). Samples were collected at 0, 6, and 16 h post-infection (hpi) and the GFP signal was quantified by flow cytometry (FACSCalibur flow cytometer; Becton Dickinson & Co, San Jose, CA, USA) on 5000 viable cells (dead cells were excluded by propidium iodide staining). The signal at 0 hpi was considered a negative signal and the percentage of GFP-positive cells was determined at 6 and 16 hpi.

### 2.8 “Click Chemistry” and MCMV DNA replication

MCMV DNA replication was detected at 16-24 hpi by incorporation of 5-ethynyl-2′-deoxyuridine (EdU) and its detection by a fluorescent azide using a Cu(I)-catalyzed [3 + 2]-cycloaddition reaction (Salic and Mitchison, 2008), as described previously (Mahmutefendić Lučin et al., 2023). The infection conditions of 1 PFU/cell block the incorporation of EdU into host cell DNA and allow visualization of replicated MCMV DNA starting at 15-16 hpi (Mahmutefendić Lučin et al., 2023). Cells treated with siRNA were infected with MCMV 48 h after transfection, and the infected cells were incubated with EdU at 16-24 hpi. The fixed and permeabilized cells were subjected to a click reaction with AF^555^ fluorescent azide, washed in PBS containing 3% BSA and incubated with the primary antibodies anti-IE1 and anti-SNX27, followed by AF^680^-conjugated anti-mouse IgG_1_ and AF^488^-conjugated anti-mouse IgG_2a_, respectively. Cells were analyzed by confocal imaging using a Leica DMI8 inverted confocal microscope, and the percentage of EdU-labeled cells was determined by quantification of IE1- and EdU-positive cells using an Olympus BX52 fluorescence microscope (DP72CCD camera, CellF software, 400x magnification).

### 2.9 Western-blot analysis

To obtain whole cell lysates (WCL), cells were lysed with RIPA lysis buffer supplemented with protease inhibitors (Roche Diagnostics GmbH, Unterhaching, Germany; Cat. No. 11697498001) and mixed with sample buffer (50% glycerol, 10% SDS, 0.05% bromophenol blue, 0.3M Tris, pH 6.8). Proteins were separated by SDS-PAGE (Bio-Rad PowerPac Universal, Hercules, CA, USA) and blotted onto a polyvinylidene difluoride membrane (PVDF-P WB membrane; Millipore, Burlington, MA, USA) at 80 V for two hours using Bio-Rad Trans-Blot Turbo Transfer System (Hercules, CA, USA). Membranes were blocked for 1 to 2 hours in 1% blocking reagent (Roche Diagnostics GmbH, Mannheim, Germany) and incubated with the appropriate primary antibody (overnight at 4°C), washed three times in T-TBS (TBS with 0.05% Tween 20; pH = 7.5) and labeled with peroxidase (POD)-conjugated secondary antibody in T-TBS containing 0.5% blocking reagent for 45-60 min. The membranes were washed again in T-TBS and the signal was detected by chemiluminescence (SignalFire [TM] Plus ECL Reagent or SignalFire [TM] Elite ECL Reagent; Cell Signaling, Cat. No. 12630S or 12757P, respectively) using Transilluminator Alliance 4.7 (Uvitec Ltd., Cambridge, UK) and ImageQuant LAS 500 (GE Healthcare Bio-Sciences AB, Upsala, Sweden). In each experiment, the expression of relevant protein(s) and β-actin as loading control, was detected at the same membrane. Quantitative analysis of the chemiluminescence signal was performed using ImageJ 1.53 software and ImageQuantTL, version 10.2., Cytiva. All values were normalized to the signal from β-actin, which was used as a loading control. First, the β-actin signals were normalized according to the following formula: Lane normalization factor = Observed actin signal for each lane/Highest observed actin signal for the blot. Second, normalized actin value was then used to normalize the experimental signal (raw signal value/normalized actin index). The following formulas were used: (1) to calculate the kinetics of host-cell protein expression during MCMV infection = Normalized experimental signal (t_x hpi_)/Normalized experimental signal (t_0 hpi_); (2) to calculate the fold change in MCMV protein expression after siRNA treatment = Normalized experimental signal (t_x siRNA hpi_)/Normalized experimental signal (t_x scr-siRNA hpi_).

### 2.10 Transfection of murine fibroblasts with pEGFP-N1-mSNX27 constructs

pEGFP-SNX27 constructs were gifts from Josef Kittler (University College London, UK): pEGFP-N1-mSNX27 (Addgene plasmid #163617; http://n2t.net/addgene:163617; RRID: Addgene_163617); pEGFP-N1-mSNX27-H112A (Addgene plasmid #163619; http://n2t.net/addgene:163619; RRID: Addgene_163619); and pEGFP-N1-mSNX27-ΔF3 (Addgene plasmid #163618; http://n2t.net/addgene:163618; RRID: Addgene_163618). For transient transfection, Balb3T3 fibroblasts were cultured on coverslips in 12- or 24-well plates and transfected with EGFP-mSNX27 constructs using Lipofectamine 3000 transfection reagent (TR) (Invitrogen, Carlsbad, CA, USA; Cat. No. L3000001) according to the manufacturer’s guidelines: Solutions containing Lipofectamine 3000 TR (1 μl) and DNA solution (1 μg plasmid DNA with 1 μl P3000 reagent) were mixed, incubated at r.t. for 10-20 min, and added dropwise to cells (70% confluent). Cells were infected with MCMV (MOI 10) 30 h after transfection, fixed 16 hpi, and permeabilized with Tween 20 (0.1%). After labeling with appropriate primary and secondary antibodies, the cells were analyzed by immunofluorescence microscopy.

### 2.11 Subcloning of pEGFP-N1-mSNX27 construct into the lentiviral vector pLIX_Kan_PstI and generation of NIH3T3-pEGFP-mSNX27 cells

EGFP-mSNX27 ORF was subcloned from the pEGFP-N1-mSNX27 plasmid (Addgene plasmid #163617; http://n2t.net/addgene:163617; RRID: Addgene_163617) into the pLIX_Kan-PstI lentiviral vector that allows doxycycline-inducible expression of the transgene (Kutle et al., 2020). In the pLIX_Kan_Pst vector, PstI and BamHI sites were used to replace the kanamycin resistance cassette (kan) with the PCR amplified EGFP-mSNX27 ORF [forward primer: 5’-CGCCTGGAGAATTGGCTGCAGTCCGCTAGCGCTACCGGA-3’ (providing PstI site) and reverse primer 5’-AAGGCGCAACCCCAACCCCGTTACTTGTACAGCTCGTCCATGC-3’] using NEBuilder HiFi DNA Assembly Cloning Kit (New England Biolabs (NEB)Mass, Ippswich, MA, USA). PstI and BamH1 restriction endonucleases were from NEB.

The constructed lentiviral plasmid pLIX-Kan-PstI_EGFP-mSNX27 was used to produce the NIH3T3-pEGFP-mSNX27 cell line with doxycycline-inducible expression of EGFP-mSNX27. For lentivirus production, 5 μg of pLIX-Kan-PstI_EGFP-mSNX27 was mixed with 10 μg of p8.91 (a gift from Simon Davis; Addgene plasmid #187441; http://n2t.net/addgene:187441; RRID: Addgene_187441) and 0.5 μg of plasmid p-CMV-VSV-G (a gift from Bob Weinberg; Addgene plasmid #8454; http://n2t.net/addgene:8454; RRID: Addgene_8454) in 1.5 ml of Optimem (Thermo Fisher Scientific, Waltham, MA, USA; Cat. No. 31-985-070) and with 41 μl of Lipofectamin 3000 in 1.5 ml of Optimem. The solution was added dropwise to 10-cm dishes containing 80-85% confluent HEK 393T cells (ATCC clone A31, ATCC CRL-3216, Manassas, VA, USA) in 10% FCS DMEM without antibiotic. The supernatants containing the lentiviruses were collected after 24, 30, and 48 h, centrifuged (5 min at 2,000 rpm), filtered (0.45 μM filter), and used for transduction of NIH3T3 cells with puromycin (2.5 μg/ml) as a selection marker. Finally, the puromycin-resistant EGFP-positive cells were additionally sorted using the FACSAria cell sorter (Becton Dickinson & Co, San Jose, CA, USA).

### 2.12 Virus growth and plaque assay

Balb3T3 fibroblasts grown in 24-well plates were treated with SNX27 siRNA or scr-siRNA for 48 h or were non-transfected. Cells were then infected with MCMV (MOI of 10) for an additional 48 and 72 h. Cell lysates and supernatants were then collected, and the production of released virions was determined using the standard plaque assay, as described previously (Štimac et al., 2021).

### 2.13 Statistical analysis and data presentation

Data comparison was performed using a two-tailed Student’s t-test when two samples were compared and one-way ANOVA analysis of variance for data with more than two experimental groups. Differences were considered significant when *p* values were < 0.05 (*p ≤ 0.05; **p < 0.01; ***p < 0.001).

## 3 Results

### 3.1 Sorting Nexin 27, sorting nexin 1, and Vps35 are recruited to membranes of the pre-AC

**Figure 1:**
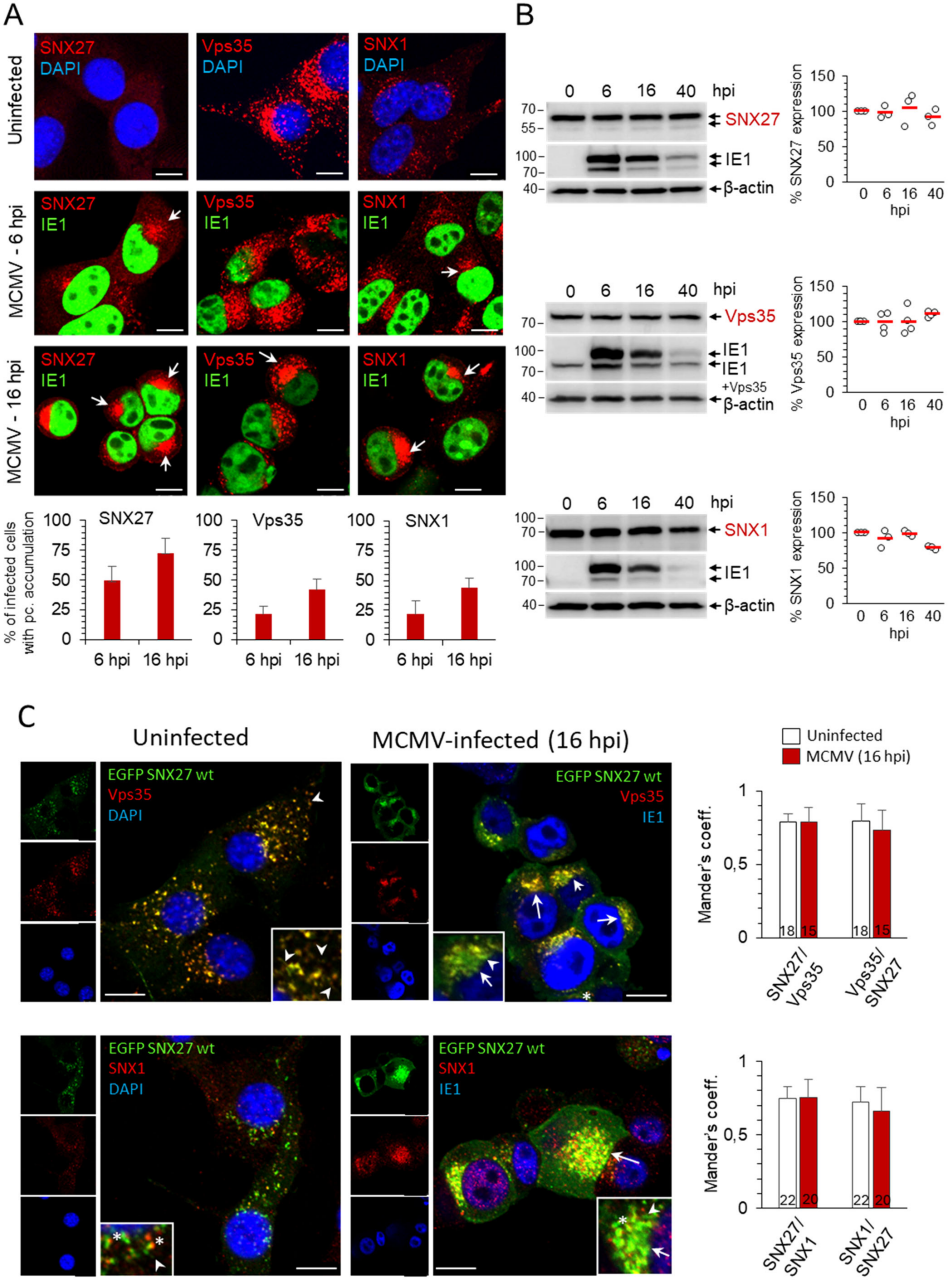
Distribution of SNX27, Vps35 and SNX1 in uninfected and MCMV-infected cells. **(A)** Balb3T3 fibroblasts were infected with Δm138-MCMV (MOI of 10) or left uninfected. Samples were stained with antibodies against SNX27, Vps35 or SNX1 (red), the pIE1 of MCMV (green), or DAPI (blue), followed by confocal imaging and quantitative analysis. The percentage of cells with pericentriolar accumulation of SNX27, Vps35 and SNX1 in MCMV-infected cells was presented as the mean±SD. Arrows indicate pericentriolar pre-AC. Bars, 10 μm. **(B)** Western blots of the expression kinetics of SNX27, Vps35 and SNX1 in the course of MCMV infection. Signals were quantified using ImageJ and expressed as a percentage of the initial expression. Shown are the individual results (empty circles) and the average (red bars) of three (SNX27 and SNX1) and four (Vps35) experiments. **(C)** Colocalization of recombinant SNX27 (EGFP-mSNX27) with endogenous Vps35 and SNX1. NIH3T3 cells expressing EGFP mSNX27 were either uninfected or infected with Δm138-MCMV (MOI of 10, 16 hpi). After fixation and permeabilization, immunofluorescence labeling with primary and appropriate secondary antibodies was performed, and cells were analyzed by confocal microscopy. Arrows indicate pericentriolar pre-AC in MCMV-infected cells; asterisks indicate endosomal partitioning of SNX1 and SNX27, and arrowheads indicate SNX27+/Vps35+/SNX1+ tubules. Mander’s coefficients (M1 and M2) on serial images were calculated to quantify colocalization. Data represents mean ± SD of 15-22 cells (number shown within the column) from three independent experiments. Bars, 10 μm.

Many cytoplasmic proteins that regulate endosomal flux are retained on membranes within the inner AC of MCMV-infected cells (Lučin et al., 2018, 2020). The retention develops gradually early in the infection, prior to viral DNA replication and expression of late viral proteins, in a structure designated as pre-AC (Taisne et al., 2019; Lučin et al., 2020). Thus, we first used confocal imaging to investigate the distribution of SNX27, Vps35 and SNX1 in the early phase of infection. In uninfected fibroblast-like cells, SNX27 was mainly dispersed in the cytosol in barely detectable punctate structures (**Figure 1A**). In MCMV-infected cells, SNX27-positive structures gradually accumulated in the perinuclear area (**Figure 1A**, indicated by arrows) of 49.5 ± 11.8% of cells at 6 hpi and 72.9 ± 11.9% of cells at 16 hpi (**Figure 1A**). This pattern was not associated with a change in the total cellular SNX27 amount (**Figure 1B**). In contrast to SNX27, Vps35, an essential component of the Retromer complex (Vps26:Vps29:Vps35), and SNX1, an essential component of ESCPE-1 that interacts with SNX27 to promote tubulation and cargo exit (Simonetti et al., 2023), were recruited to clearly visible cytoplasmic membrane structures of uninfected cells (**Figure 1A**), consistent with their localization at endosomes. Similar to SNX27, Vps35- and SNX1-positive membranous organelles concentrated in the perinuclear area of MCMV-infected cells at 6 and 16 hpi (**Figure 1A**, arrows), and MCMV infection did not alter their expression (**Figure 1B**).

As expected from the data from the literature, Vps35 and SNX1 colocalized mainly on endosomes of uninfected cells (**Supplementary Figure S1**) as well as at membranous structures within the inner pre-AC of MCMV-infected cells (**Supplementary Figure S1**). Since endogenous SNX27 was barely detectable by Abs at distinct membrane elements of uninfected cells, we established the NIH3T3 cell line with inducible expression of EGFP-SNX27. In NIH3T3 cells, which are more suitable for transfection, the detection of endogenous SNX27 on membrane elements by Abs was as low as in Balb3T3 cells. After induction, EGFP-SNX27 was recruited to Vps35-positive and SNX1-positive structures (**Figure 1C**). These data suggest that both Retromer- and ESCPE-1-associated functions operate in fibroblast-like cells used for infection, consistent with their known localization at EEs and their distribution in recycling carrier-generating EE microdomains as components of large (SNX27:Retromer:ESCPE-1) or smaller (SNX-BAR or SNX27:Retromer) complexes (Lauffer et al., 2010; Cullen and Steinberg, 2018). In the fully developed pre-AC of MCMV-infected cells (at 16 hpi), SNX27, Vps35, and SNX1 localized in the inner pre-AC (**Figures 1C**, **Supplementary Figure S1**, arrows) and exhibited a similar level of colocalization as in uninfected cells (**Figure 1C**, **Supplementary Figure S1**, **Supplementary Table S1**). A similar expression pattern to SNX27 was observed for SNX2 (**Supplementary Figure S2**), a component of ESCPE-1 that is redundant to SNX1 (Simonetti et al., 2023).

Overall, reorganization of the membrane system during the E phase of MCMV infection is also associated with retention of SNX27, ESCPE-1, and Retromer suggesting that the expanded membranes within the inner pre-AC are capable of cargo retrieval and tubulation. Essentially, the same results were obtained in the NIH3T3 cell line with inducible expression of EGFP-SNX27 (**Figure 1C**) as in Balb3T3 cells (**Figure 1A**, **Supplementary Figure S1**), since infection in Balb3T3 cells increased the visualization of endogenous SNX27 through its retention on membranous structures. Thus, to study endogenous expression conditions, we performed further experiments on infected Balb 3T3 cells.

### 3.2 Suppression of SNX27, Retromer and ESCPE-1 prevents pericentriolar accumulation of Rab10 membranes in the pre-AC but not dislocation of the Golgi

**Figure 2:**
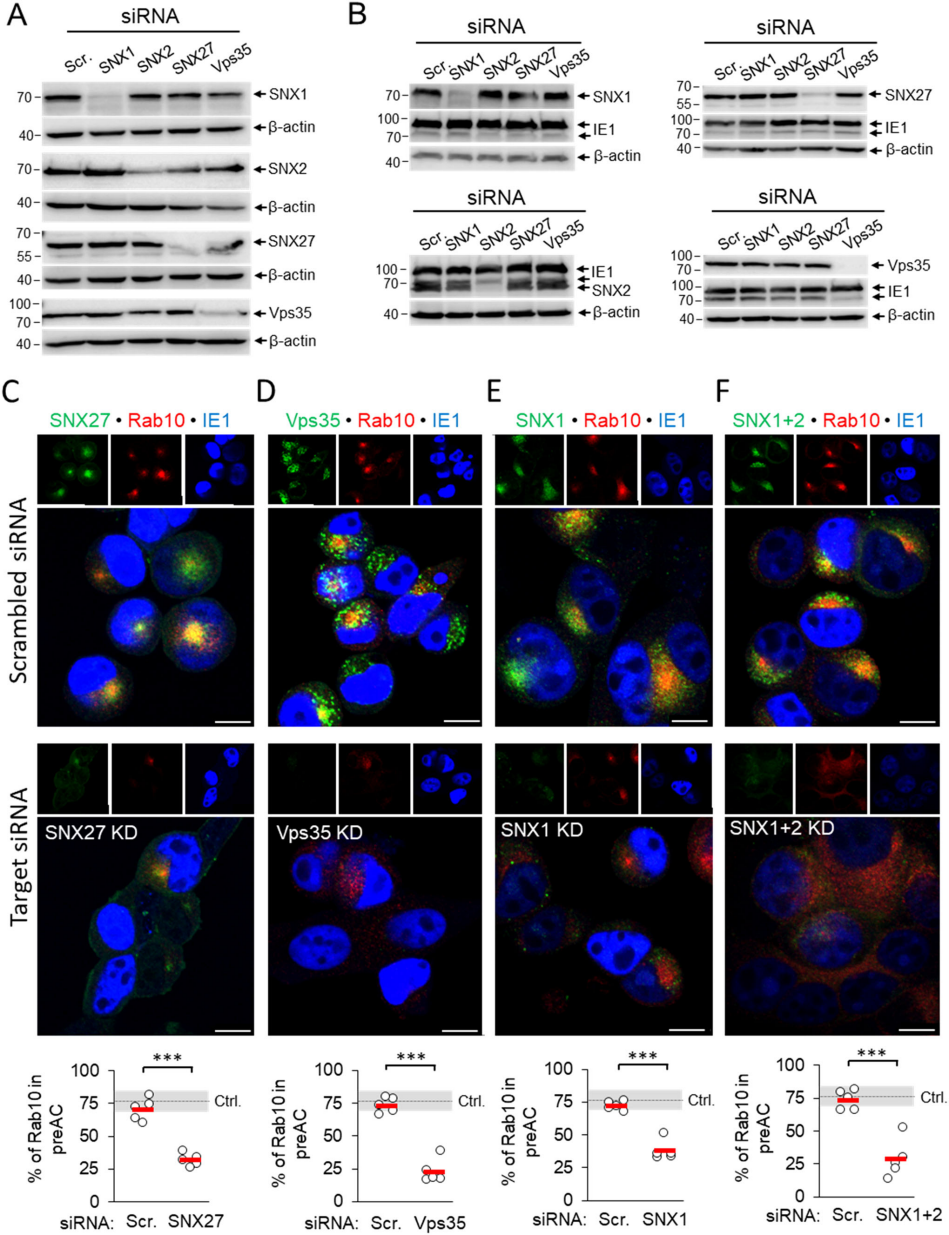
siRNA depletion of SNX27, Vps35, SNX1 and SNX1 + 2 prevents pericentriolar accumulation of Rab10 in the early phase of MCMV infection. **(A)** Balb3T3 fibroblasts were transfected with scrambled siRNA or siRNA for SNX27, Vps35, SNX1, and SNX1 + 2. After 48 h, the expression of depleted proteins was detected by Western blot using β-actin as a loading control. **(B)** siRNA-treated cells (48 h after transfection) were infected with Δm138-MCMV (MOI of 10), and at 16 hpi the expression of depleted proteins was analyzed by Western blot. The pIE1 expression served as a marker for infection, and β-actin was used as a loading control. **(C-F)** Infected cells (16 hpi) transfected with either control siRNA or siRNA for SNX27, Vps35, SNX1 and SNX1 + 2 were stained with antibodies for either SNX27 **(C)** or Vps35 **(D)** or SNX1 **(E)** or SNX1 + 2 **(F)** to visualize these proteins (green fluorescence) combined with antibodies for Rab10 (red fluorescence) and pIE1 (blue). The stained cells were analyzed by confocal imaging, and the percentage of cells expressing pericentriolar Rab10 accumulation was determined by epifluorescence image analysis. The data show individual results from five independent experiments. Ctrl., control level (mean ± SD) from 18 independent experiments. Mean values are shown as red bars, and statistical significance was determined using a two-tailed Student t-test (***p < 0.001). Bars, 10 μm.

The development of the pre-AC is initiated at 4-6 hpi by the simultaneous displacement of the Golgi and the expansion of the EE, ERC, and TGN membranes within the inner pre-AC (Karleuša et al., 2018; Lučin et al., 2020). The accumulation of Rab10 is a hallmark of this reorganization and represents the earliest event that can be used for monitoring pre-AC development by immunofluorescence imaging, together with markers of the Golgi. Therefore, to investigate the contribution of SNX27:Retromer:ESCPE-1-associated functions in pre-AC development, we monitored pericentriolar Rab10 accumulation after knockdown of SNX1, SNX2, SNX27, and Vps35 expression with siRNA. After successful suppression of these proteins in uninfected cells (**Figures 2A**, **Supplementary Figure S3**), cells were infected with MCMV for 16 h. The knockdown state was confirmed by simultaneous Western blot detection of these proteins and viral pIE1 (**Figure 2B**) and cell viability by flow cytometry (**Supplementary Table S2**). None of the knockdown procedures resulted in the pericentriolar accumulation of endogenous Rab10 in uninfected cells (**Supplementary Figure S3**). Both untreated and control scr-siRNA-treated cells developed pericentriolar accumulation of Rab10 in MCMV-infected cells, indicating that the transfection procedure and the introduction of foreign irrelevant RNA do not affect membrane reorganization during MCMV infection (**Figures 2C-F**). By contrast, knockdown of SNX27 (**Figure 2C**), Vps35 (**Figure 2D**), and SNX1 (**Figure 2E**) significantly reduced the percentage of cells with pericentriolar Rab10 accumulation. A similar effect was observed after the simultaneous knockdown of SNX1 and SNX2 (SNX1 + 2, **Figure 2F**), as they act redundantly in the SNX-BAR (ESCPE-1) coat complex; however, depletion of SNX2 alone had no effect (**Supplementary Figure S4A**). Collectively, these data suggest that SNX27:Retromer:ESCPE-1 complexes are required for Rab10-associated membrane reorganization events in the pre-AC.

Suppression of the SNX27:Retromer:ESCPE-1 complexes did not prevent compaction and displacement of the Golgi into the ring-like configuration that forms the outer pre-AC, as demonstrated by visualization of the cis-Golgi marker GRASP65. In untreated cells, SNX27-, SNX1/2-, and Vps35-positive membranous structures localized mainly within the GRASP65-positive ring representing the inner pre-AC (**Supplementary Figure S5**, arrows), whereas suppression of SNX27, Vps35, SNX1, and SNX1+2 had no effect on the distribution of GRASP65-positive membranes in uninfected cells (**Supplementary Figure S6**) and did not prevent Golgi compaction in MCMV-infected cells at 16 hpi (**Supplementary Figures S5, S4B**).

### 3.3 Pericentriolar accumulation of Rab10 membranes in the inner pre-AC requires a functional SNX27 F3-FERM subdomain

**Figure 3:**
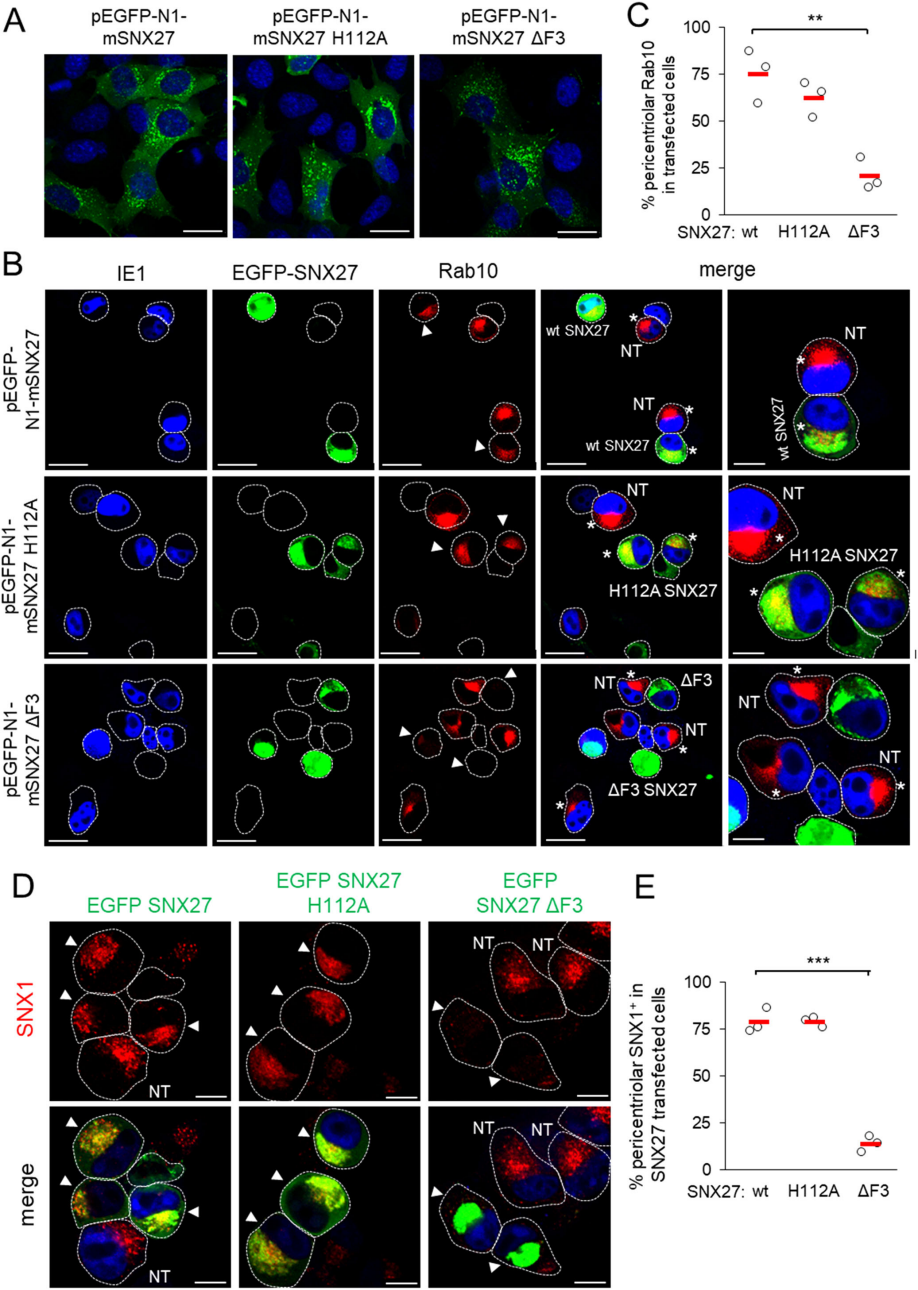
The F3-FERM subdomain of SNX27 is required for pericentriolar Rab10 and SNX1 accumulation in the pre-AC. **(A-E)** Balb3T3 fibroblasts were transfected with pEGFP-N1-mSNX27, pEGFP-N1-mSNX27 H112A, or pEGFP-N1 SNX27 ΔF3 plasmids. After 30 h, the cells were fixed **(A)** or infected with Δm138-MCMV (MOI of 10) for a further 16 h before fixation **(B-E)**. Subsequently, the permeabilized cells were labeled with anti-Rab10 or anti-SNX1 antibodies (red), anti-IE1 antibodies (blue, infected cells) or DAPI (blue, uninfected cells) and analyzed by confocal microscopy. **(B)** Asterisks represent cells with pericentriolar Rab10, and **(B, D)** arrowheads represent transfected cells. **(C, E)** The graphs show the percentage of MCMV-infected cells (IE1-positive) with pericentriolar accumulation of Rab10 **(C)** or SNX1 **(E)**, determined in three independent experiments (empty circles), and the mean values (red bars). Statistical significance was determined using a two-tailed Student t-test (***p < 0.001; **p < 0.01). NT, non-transfected. Bars, 25 μm and 10 μm **(B)**, and 10 μm **(D)**.

To gain further insight into the role of SNX27:Retromer:ESCPE-1 in the development of the Rab10-associated components of the pre-AC, we investigated the role of cargo retrieval and complex formation of SNX27 via its PDZ domain and F3-FERM domain, respectively. The PDZ domain recognizes cargo and interacts with Vps26 in the Retromer (Temkin et al., 2011; Cullen and Steinberg, 2018). The F3-FERM subdomain binds SNX1 or SNX2 to form full SNX27:Retromer:ESCPE-1 complexes (Yong et al., 2021; Simonetti et al., 2022), regulates interactions with endosomal cargoes, and serves as a scaffold for signaling complexes (Chandra et al., 2021). We transfected cells to overexpress wt-SNX27 (pEGFP-N1-mSNX27), SNX27 with the point mutation H112A that disables cargo but not Retromer binding (Gallon et al., 2014) to the PDZ domain (pEGFP-N1-mSNX27 H112A), and SNX27 with a deleted F3-FERM subdomain (pEGFP-N1-mSNX27 ΔF3), in which the interaction of SNX27 with SNX1/SNX2 and signaling complexes is disabled (Halff et al., 2019). In uninfected cells, wt-SNX27 and the mutants SNX27 H112A and SNX27 ΔF3 showed vesicular patterns (**Figure 3A**) that corresponded to EEs, as previously described (Halff et al., 2019; Simonetti et al., 2022). After MCMV infection (16 hpi) of transfected cells, all three forms of SNX27 accumulated in the pericentriolar region of the pre-AC (**Figure 3B**). In wt-EGFP-SNX27- and EFGP-SNX27 H112A-transfected cells, Rab10 accumulated in the pericentriolar region (**Figure 3B**, arrowheads) to a comparable degree as in non-transfected cells (**Figure 3B**, NT), whereas in cells overexpressing the EGFP-SNX27-ΔF3 Rab10 enrichment was low or absent (**Figure 3B**, indicated by arrowheads at the bottom row). These data suggest that the F3-FERM subdomain is required for downstream events associated with Rab10 accumulation. Accordingly, SNX1 accumulated in pre-AC of cells transfected with wt-EGFP-SNX27 or EGFP-SNX27 H112A, but very little in cells expressing EGFP-SNX27 ΔF3 (**Figures 3D, E**). In EGFP-SNX27 ΔF3-expressing cells, SNX1 was dispersed in the cytosol and occasionally exhibited scattered dots (**Figure 3D**). These data are consistent with the activity of the F3-FERM subdomain in recruiting the ESCPE-1 proteins SNX1/SNX2, which are required for the expansion of the subset of Rab10-associated membranes within the inner pre-AC. As Rab10 is an essential component of tubular endosomes (Etoh and Fukuda, 2019), these data suggest that SNX27:ESCPE-1 complexes are required for the initiation of tubulation and Rab10 recruitment at very early stages of tubule biogenesis. Moreover, these data indicate that the binding of cargo proteins to the PDZ domain is not required for Rab10-associated membrane reorganization.

### 3.4 Depletion of SNX27:Retromer:ESCPE-1 alters progression of the MCMV replication cycle

**Figure 4:**
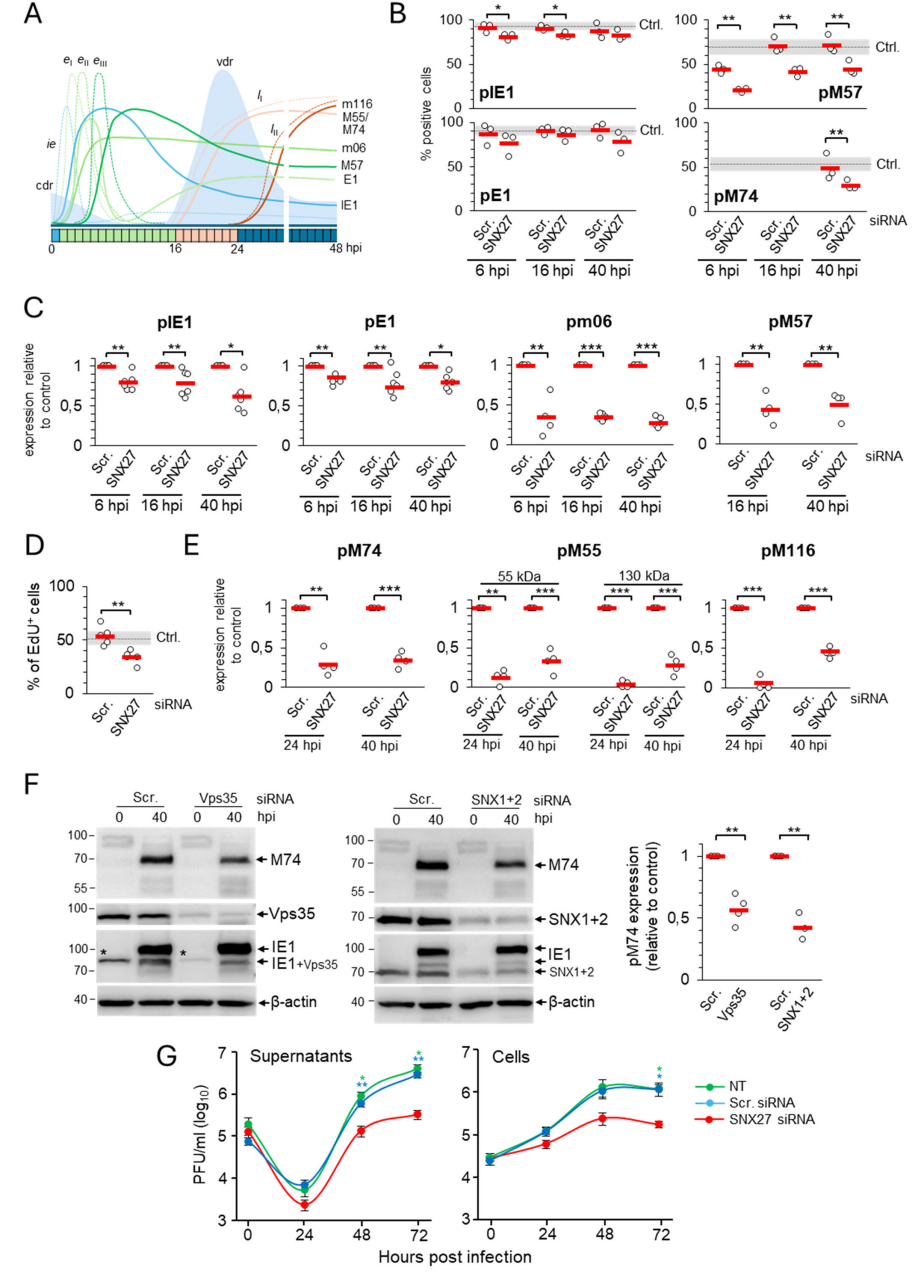
Depletion of SNX27 inhibits progression through the productive infection. Balb3T3 fibroblasts were transfected with scrambled (Scr.) or SNX27 siRNAs. After 48 hours, cells were infected with Δm138-MCMV (MOI of 10) and analyzed at 6, 16, 24, and 40 hpi by immunofluorescence and Western blot. **(A)** Schematic presentation of the viral replication cycle program. The available antibody reagents against seven MCMV-encoded proteins can distinguish immediate early (*ie*), three temporal classes of early (*e*_I_, *e*_II_, and *e*_III_), and two temporal classes of late (*l*_I_ and *l*_II_) gene expression. The kinetics of the temporal classes of transcription are shown by dotted lines, and protein expression by full lines using different colour codes. The temporal effect of MCMV infection on cellular DNA replication (cdr) and the timing of viral DNA replication (vdr) are shown in the light-blue area. **(B)** Percentage of cells expressing viral proteins at 6, 16, and 40 hpi. Triple-stained (viral protein, SNX27, and DAPI) immunofluorescence samples were captured by confocal imaging (**Supplementary Figures S9, S10A**) and quantitatively analyzed using epifluorescence microscopy. Open circles represent data from three independent experiments, and red bars show the average value. Ctrl., control level (mean ± SD) determined in independent experiments (n=25 for pIE1, n=22 for pE1, n=12 for pM57, and n=8 for pM74). **(C)** Quantification of *ie, e*_I_, *e*_II_, and *e*_III_ viral protein expression by Western blot. Viral proteins pIE1, pE1, pm06, and pM57 were analyzed simultaneously (at the same membrane) with SNX27 and actin (**Supplementary Figure S7**). The signals were quantified, viral protein expression in SNX27-depleted cells normalized to actin expression, and the result was shown as a relative to control (scr-siRNA-treated cells). Results are presented as fold changes compared to scr-siRNA in the respective kinetics (means ± SD) by empty circles, and averages by red bars. **(D)** EdU labeling of viral DNA replication. Cells transfected with Scr. and SNX27 siRNA were infected and labeled 16-24 hpi with 10 μM EdU, followed by staining with antibodies against SNX27 and pIE1, and visualization of EdU-labeled DNA with the click reaction. Shown is the percentage of EdU-positive cells, whereas the triple-labeled images are shown in **Supplementary Figure S10B**. The empty circles show the values in an independent experiment, and the red bars show the mean value. Ctrl., control level (mean ± SD) determined in 15 independent experiments. **(E)** Quantification of *l*_I_ and *l*_II_ viral protein expression (pM55, pM74, and pm116) was performed by Western blot as described in **(C)**. The representative images of Western blots are shown in **Supplementary Figure S7**. Two forms of pM55 (55 and 130 kDa) were analyzed. **(F)** Effect of depletion of Vps35 and SNX1+2 on the expression of the late protein pM74. Shown are the representative Western blots of pM74, Vps35, and SNX1+2 expression in Vps35- and SNX1+2-depleted cells at 40 hpi, respectively. The asterisk represents remnants of Vps35 detection overlaying the lower band of pIE1. The pM74 signals were quantified, normalized to actin, and shown as a fold change compared to the scr-siRNA (empty circles). The average values are shown as red bars. **(G)** Depletion of SNX27 reduces virus production. Single-step growth kinetics in non-transfected (NT), scr-siRNA- or SNX27 siRNA-transfected Balb3T3 cells after infection with Δm138-MCMV (MOI of 10). Supernatants and cells were harvested at indicated times post-infection, and frozen at -80°C. After two rounds of freeze/thaw, the virus was quantitated by the plaque assay on MEFs. Data represents log_10_ infectious units/mL of sample, and error bars indicate standard errors of the means of six biological replicates. Statistical significance was determined using a two-tailed Student t-test **(B-F)** or one-way ANOVA analysis **(G)**. The colour of the asterisks **(G)** denotes the statistical difference of NT (green) and Scr. (blue) from SNX27. ***p < 0.001; **p < 0.01; *p < 0.05.

Previous experiments show that depletion of SNX27 and SNX27-associated complexes abolishes an important step in the MCMV replication cycle that contributes to membrane reorganization and the formation of the pre-AC. Although all immunofluorescence experiments shown above included pIE1 expression as a control of infection at the cell population level, we consistently observed a degree of attenuation of the pIE1 signal by fluorescence microscopy. Thus, to clarify the observed effects of SNX27:Retromer:ESCPE-1 depletion, we performed a more thorough analysis of the progression of the MCMV replication cycle. We analyzed seven MCMV-encoded proteins and viral DNA replication to monitor various stages of the MCMV replication cycle (Lacaze et al., 2011; Marcinowski et al., 2012; Ružić et al., 2022; Mahmutefendić Lučin et al., 2023), as schematically illustrated in **Figure 4A**.

The percentage of cells expressing pIE1 and pE1, key indicators for entry into the E phase of infection, was similar in cells transfected with scr-siRNA (**Figures 4B**, **Supplementary Table S3**) and untreated cells (Lučin et al., 2020). However, infection of SNX27-depleted cells consistently resulted in a small but significant decrease in the percentage of cells expressing pIE1 (**Figure 4B**, **Supplementary Table S3**). Western blot analysis of pIE1 and pE1 also showed a consistent decrease in their expression in the E (6 hpi), late-E (16 hpi), and L (40 hpi) phases of infection (**Figures 4C**, **Supplementary Figure S7**, **Supplementary Table S4**). As these data suggest that SNX27-dependent functions are already required in the E phase of infection, we extended our analysis to all necessary components of SNX27:Retromer:ESCPE-1 complexes using well-established MCMV-GFP infection and flow cytometric quantification of the viral protein expression program (Angulo et al., 2000). This analysis confirmed a mild reduction of the fluorescence intensity and percentage of GFP-positive cells after SNX27-depletion (**Supplementary Figures S8A, B**), suggesting that SNX27-associated functions contribute to the control of virus replication prior to the establishment of the Rab10-associated part of pre-AC and that expression of E proteins that mediate membrane system reorganization and pre-AC development appears to be sufficient, as the Golgi displacement also occurs in SNX27-depleted cells (**Supplementary Figure S5**).

As CMV gene expression kinetics can be divided into at least seven temporal classes (Rozman et al., 2022), we extended the analysis of the effects of SNX27 suppression on the expression of pm06 and pM57. These two MCMV proteins are expressed with delayed early kinetics after the first set of E genes at 2-3 and 5-6 hpi, respectively, suggesting two distinct temporal classes in MCMV-infected cells (**Figure 4A**). Immunofluorescence (**Figure 4B**) and Western blot analysis (**Figure 4C**, **Supplementary Figure S7**, **Supplementary Table S4**) showed a substantial decrease in the expression of pm06 and pM57 in SNX27-depleted cells, already in the E phase of infection (6 hpi) as well as at later time points (16 and 40 hpi). Immunofluorescence analysis of pM57 showed a reduction in the number of expressing cells (**Figures 4B**, **Supplementary Table S3**), associated with reduced accumulation of pM57 in nuclear pre-replication centers at 6 and 16 hpi (**Supplementary Figure S10**, arrowheads) and limited pM57 condensation in nuclear replication centers at 40 hpi (**Supplementary Figure S10**, arrows). The effect of SNX27 depletion on pM57, the ssDNA-binding protein involved in viral DNA synthesis (Strang et al., 2012) and viral gene transcription (Chang et al., 2011), may have an effect on viral DNA replication. Therefore, we next examined the effect of SNX27 depletion on viral DNA replication using 5-ethynyl-2-deoxyuridine (EdU) labeling (Strang et al., 2012; Mahmutefendić Lučin et al., 2023). This protocol allows visualization of replicated viral DNA in the nucleus of the infected cell at 16-24 hpi, as MCMV infection completely blocks host cell DNA replication in the E phase of infection at 6-12 hpi (**Figure 4A**, Mahmutefendić Lučin et al., 2023). As expected, EdU labeling at 16-24 hpi visualized replicated viral DNA in 53.8 ± 8.78% of cells transfected with scr-siRNA (**Figures 4D**, **Supplementary Figure S10B**), which is comparable to untreated cells (Mahmutefendić Lučin et al., 2023). In SNX27-depleted cells, the number of cells showing an EdU-labeled signal in the nucleus was reduced to 33.8 ± 6.18% (**Figure 4D**). These data suggest that SNX27-associated functions contribute to the overall capacity of the cell to replicate viral DNA and, subsequently, the expression of viral L genes.

To assess the expression of L genes, we examined the expression of pM55 and pM74, two late glycoproteins that localize in the AC and form the virion envelope (Kattenhorn et al., 2004; Lučin et al., 2020), and pM116.1, an abundant L protein that localizes in the AC but is not integrated into the virion particles (Ružić et al., 2022). As in untreated cells (Lučin et al., 2020), pM74 perinuclear cytoplasmic staining was detected in 49.05 ± 14.8% of scr-siRNA-transfected cells at 40 hpi (**Figures 4B**, **Supplementary Figure S11**, **Supplementary Table S3**). In SNX27-depleted cells, the percentage of pM74-positive cells was reduced to 29.07 ± 5.84% expressing cells (**Figure 4B**, **Supplementary Table S3**), resulting in the reduction of pM74 expression to 30% of the control level at 24 and 40 hpi (**Figures 4E**, **Supplementary Figure S7**, **Supplementary Table S4**). In addition to pM74, 55 kDa and 130 kDa forms of pM55 were reduced to approximately 15-30% of the control level after 40 hpi in SNX27-depleted cells (**Figures 4E**, **Supplementary Figure S7**, **Supplementary Table S4**). A similar observation was made for pM116.1, which was absent at 24 hpi and reduced to 45% of the control level at 40 hpi (**Figures 4E**, **Supplementary Figure S7**; **Supplementary Table S4**). These data demonstrate that depletion of SNX27 reduces the expression of viral late proteins, including viral glycoproteins that form the virion envelope.

To distinguish whether the observed effects of SNX27 depletion are associated with its contribution to the SNX27:Retromer:ESCPE-1 complexes, we examined the effect of Vps35 and SNX1 + 2 depletion on the expression of pM74 in the L phase of infection. Western blot analysis showed that the expression of pM74 was significantly reduced in both Vps35- and SNX1+2-depleted cells at 40 hpi compared to scr-siRNA-treated cells (**Figures 4F**), suggesting that the observed effects on the expression of viral proteins are associated with the function of the SNX27:Retromer:ESCPE-1 complexes.

### 3.5 Depletion of SNX27 reduces the production and release of infectious virions

Inhibition of the Rab10-associated membranous events in the inner pre-AC, viral DNA replication, and expression of late genes in SNX27-depleted cells suggest that SNX27-associated processes are required for virion assembly and release. Therefore, we next investigated the production and release of infectious virions in SNX27-depleted cells. Nontransfected Balb3T3 cells and cells transfected with control or SNX27 siRNAs were infected with 1 PFU/cell and the amount of infectious viral particles was determined at 48 and 72 hpi using a standard plaque assay. Cells treated with scr-siRNA released similar amounts of infectious particles as untreated cells (**Figure 4G**). As expected, SNX27-depleted cells released 7- and 9-fold fewer virions after 48 and 72 hpi, respectively (**Figure 4G**). A similar pattern was observed when cellular lysates (i.e., the presence of intracellular virions) were analyzed (**Figure 4G**), suggesting that the lower release of virions is related to the reduced assembly capacity rather than the inability to release assembled virions. These data confirm that SNX27-associated processes are essential in virion biogenesis.

### 3.6 The contribution of SNX27 to the progression of MCMV replication cycle requires a functional F3-FERM subdomain

**Figure 5:**
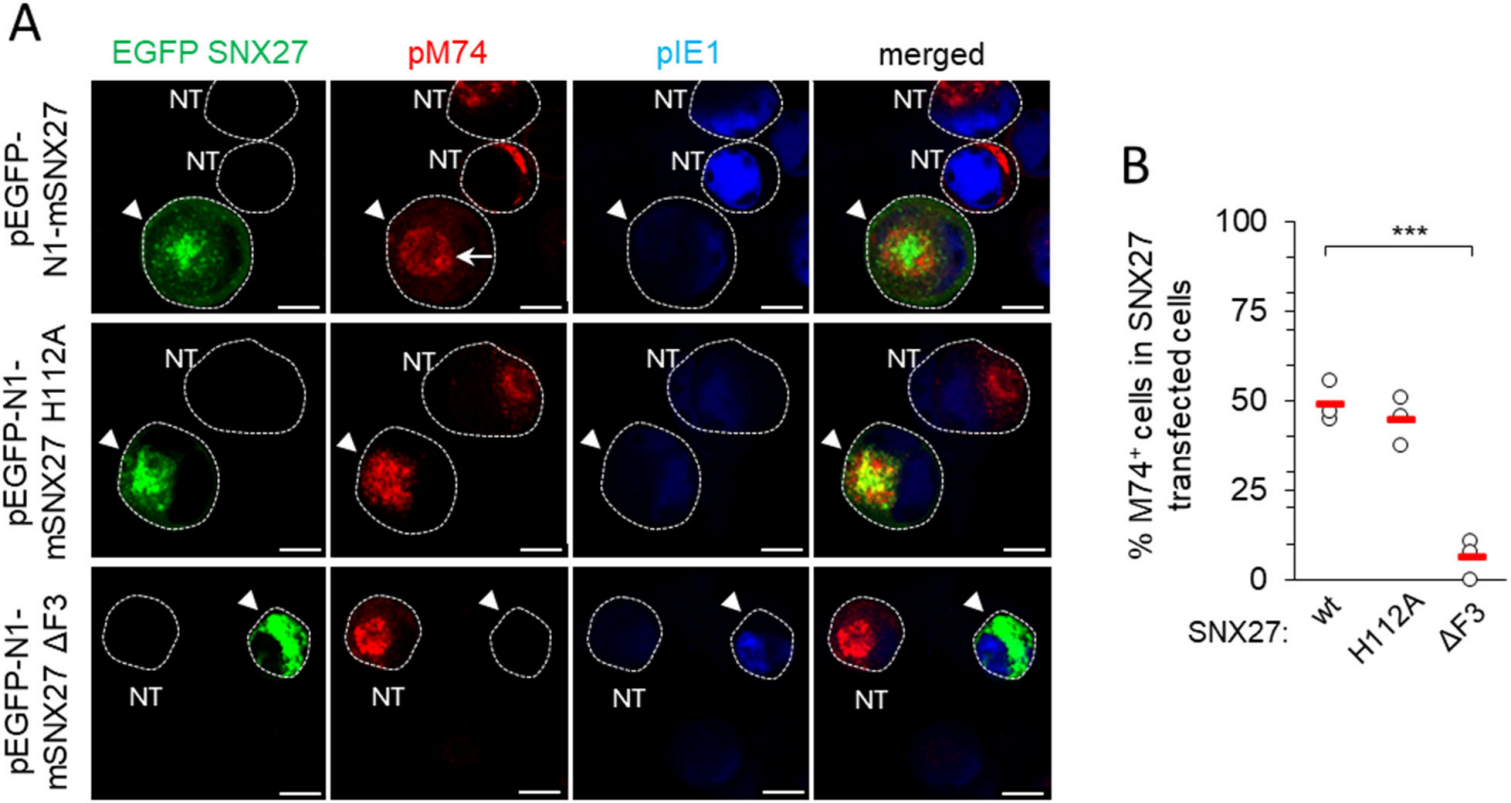
Expression of pM74 after MCMV infection of SNX27 wt, SNX27 H112A and SNX27 ΔF3 overexpressing cells. **(A)** Immunofluorescence analysis of transfected and non-transfected cells. Cells were transfected with EGFP SNX27 wt, EGFP SNX27 H112A and EGFP SNX27 ΔF3 and 48 hours later infected with Δm138-MCMV at an MOI of 10. 48 hours after infection, the cells were stained for the proteins pM74 (red) and pIE1 (blue). The cell boundaries are indicated by fine dashed lines. Arrowheads indicate transfected cells and arrows indicate ring-shaped pM74 expression. Bars, 10 μm. **(B)** The percentage of SNX27-transfected and MCMV-infected cells expressing pM74 was determined in three independent experiments (empty circles). The red bars represent the mean value. Statistical significance was determined using a two-tailed Student t-test (*** p < 0.001). NT, non-transfected.

Downstream effector functions of the SNX27 are associated with the retrieval functions mediated by its PDZ and F3-FERM domains, including PDZ-mediated recruitment of the Retromer and F3-FERM-mediated recruitment of the SNX1 or SNX2 component of ESCPE-1 (Seaman, 2018). To elucidate the contribution of SNX27 domains, we monitored the expression and subcellular distribution of pM74 by immunofluorescence and confocal imaging in pEGFP-N1-mSNX27, pEGFP-N1-mSNX27 H112A, and pEGFP-N1-mSNX27 ΔF3 transfected and MCMV-infected cells. A ring-shaped perinuclear expression of pM74 (**Figure 5A**, arrow, top row) was detected in 48.96 ± 5.69% wt-SNX27 transfected cells after 48 h of MCMV infection (**Figure 5B**), similar to previous studies with untreated cells (Lučin et al., 2020). EGFP SNX27 wt was concentrated within the pM74 ring, consistent with its accumulation in the inner AC (**Figure 5A**). Infection of cells overexpressing SNX27 with the H112A mutation resulted in a similar number of pM74-expressing cells (44.76 ± 6.81% of transfected cells; **Figure 5B**), including a similar ring-shaped distribution of pM74 and accumulation of EGFP SNX27 H112A in the inner AC (**Figure 5A**, middle row). However, overexpression of the SNX27 mutant lacking the F3-FERM subdomain almost completely prevented the expression of pM74 after MCMV infection, resulting in only 6.13 ± 5.54% pM74-positive transfected cells (**Figure 5A**, bottom row, and **5B**). These data suggest that the mechanism driving MCMV gene expression does not rely on PDZ domain-mediated recruitment of signaling receptors but rather on SNX27:Retromer:ESCPE-1 complex formation, as the H112A mutation does not prevent association with Retromer. The complex formation also requires a functional F3-FERM domain that binds ESCPE-1, consistent with the effect of SNX1 + 2 depletion on pM74 expression shown above (**Figure 4F**).

## 4 Discussion

**Figure 6:**
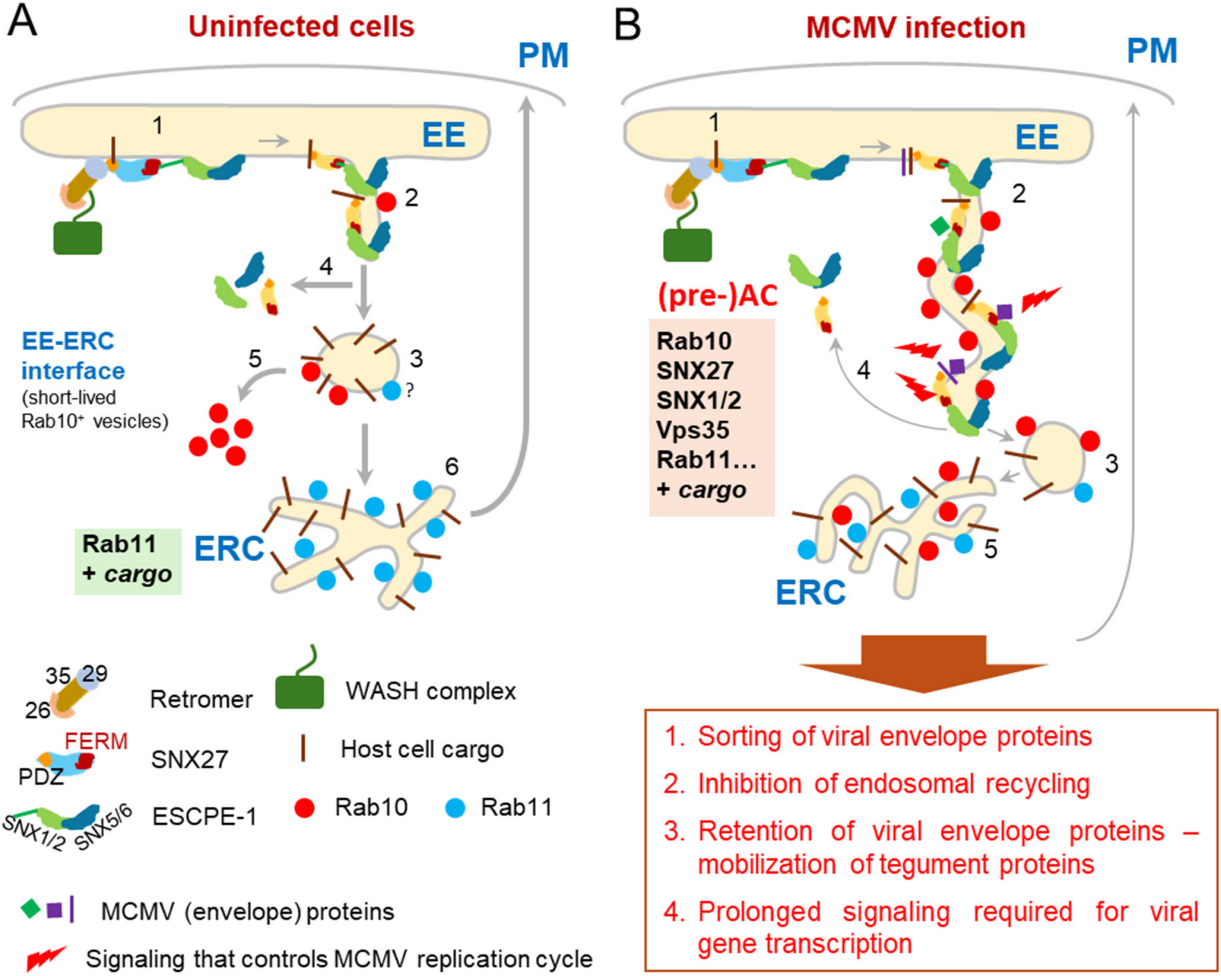
A model showing the role of the SNX27:Retromer:ESCPE-1 complex in the formation of AC in the early phase of MCMV infection. **(A)** In uninfected cells, the SNX27:Retromer:ESCPE-1 pathway involves cargo molecule recognition and recruitment of the Vps35 Retromer component by different parts of the SNX27 PDZ domain, followed by recognition of the SNX1/2-SNX5/6 (ESCPE-1) heterodimer by the SNX27-F3-FERM subdomain (1). ESCPE-1 can also recognize additional cargo (not shown). This process allows mobilization of actin by WASH1 (not shown), initiation of tubulation at the endosomal membrane, and transient binding of Rab10, which is required for tubule growth (2) and their fission into transport carriers (3) that release SNX27:Retromer:ESCPE-1 components (4) and Rab10 (5). The transport carriers migrate to the subset of ERC membranes to further sort and recycle the cargo to the PM, which is known as the slow recycling pathway (6). **(B)** The initial process of EE tubule formation (1) is functional in MCMV-infected cells. SNX27:Retromer:ESCPE-1 components and Rab10 are recruited to membranes. However, the mechanism limiting tubule growth is blocked and the development of transport carriers is inhibited (3), leading to the formation of elongated tubules (2), which retain cargo and SNX27:Retromer:ESCPE-1 components (4), resulting in reduced supply to the ERC and subsequently a reduced ERC recycling rate (5). Elongated tubules can concentrate CMV envelope proteins and serve as membranes for secondary envelopment. In addition, elongated tubules with retained SNX27:Retromer:ESCPE-1 and viral glycoproteins can serve as signalosomes by retaining or recruiting signaling proteins and establishing a signaling cascade that influences the entire MCMV replication cycle.

In this study, we show that SNX27:Retromer:ESCPE-1 complexes are required for expansion of subset of tubular membranes within the pre-AC and the progression of the viral replication cycle in CMV-infected cells. Their components are over-recruited in the inner region of the AC, a site of extensive accumulation and expansion of EEs, ERC and TGN membrane compartments. A representative sign of this expansion is the accumulation of membranes bearing Rab10, indicating tubulation at the EE-ERC interface. Depletion of SNX27, Vps35 (essential Retromer component) and SNX1+2 (essential components of ESCPE-1) prevents the accumulation of Rab10-positive membranes, suggesting that the recruitment of SNX27:Retromer:ESCPE-1 precedes the expansion of Rab10-positive membrane intermediates. Furthermore, SNX27:Retromer:ESCPE-1-associated functions are required for efficient progression of the MCMV replication cycle, as its depletion decreases the expression of virus-encoded E proteins, reduces viral DNA replication, and subsequently decreases the expression of L proteins, including viral structural proteins. The observed contributions of SNX27 require a functional F3-FERM subdomain, but not the cargo retrieval region of its PDZ domain. Thus, our data suggest that membrane reorganization driven by the SNX27:Retromer:ESCPE-1 complexes, likely tubulation, is involved in the control of viral gene expression. Based on these data, our previous studies on MCMV and studies of others on HCMV, we propose that SNX27:Retromer:ESCPE-1 complexes are involved in the establishment of a part of the inner pre-AC and additionally in the establishment of a signaling platform in CMV-infected cells (**Figure 6**).

Numerous host cell factors associated with membrane tubulation accumulate within the inner pre-AC, indicating the expansion of tubular membranes. As very little Rab10 is associated with the membranes of uninfected cells, the accumulation of Rab10 early in MCMV infection is a hallmark of this process and its pericentriolar accumulation at 5-6 hpi indicates expansion of intermediates between the EEs and the ERC (Karleuša et al., 2018; Lučin et al., 2020; Štimac et al., 2021). Rab10 can also be activated at the ER (Babbey et al., 2006; Liu et al., 2013), but the ER is excluded from the inner pre-AC of CMV-infected cells (Sanchez et al., 2000; Das and Pellett, 2011; Lučin et al., 2020). Thus, the main site of Rab10 activation is Rab5-positive EEs, where Rab5 and Rab10 form a cascade (Liu et al., 2018; Liu and Grant, 2015). In uninfected cells, activation of Rab10 drives the development of tubular recycling endosomes (TREs) (Etoh and Fukuda, 2019; Farmer et al., 2021) which sort and recycle clathrin-independent endocytic (CIE) cargo to the PM (Xie et al., 2016). In most cells Rab10 is rapidly cleared from transport intermediates after scission of tubular extensions into endocytic carriers and released into the cytoplasmic fraction and therefore, due to the short residence time, very little Rab10 can be detected at the membranes by immunofluorescence (Babbey et al., 2006). In MCMV-infected cells Rab10 remains associated with the membranes and produces a significant signal that appears as pericentriolar over-recruitment, a site of TRE or ERC membrane accumulation. The pericentriolar accumulation of several other host cell factors, such as MICAL-L1 and EHBP1 (Lučin et al., 2020), which associate with Rab10 at TREs (Xie et al., 2016; Etoh and Fukuda, 2019; Farmer et al., 2021), support the conclusion of TRE expansion and delayed maturation within the inner AC. The development of these expansions correlates with the retention of CIE cargo, as has been demonstrated for MHC-I proteins within EEs and their inhibited recycling in MCMV- (Tomaš et al., 2010; Lučin et al., 2015; Karleuša et al., 2018) and HCMV-infected cells (Zeltzer et al., 2018). In addition to Rab10-positive membranes in the inner pre-AC, CMV infection also expands other tubular membranes originating from the EE system (Lučin et al., 2020; Pavišić et al., 2021),

Here we show that SNX27:Retromer:ESCPE-1 complexes are also retained at the inner pre-AC membranes and are essential for the expansion of Rab10-positive membranes (**Figure 6**). This is consistent with the known role of these complexes in CIE cargo retrieval and tubulation at EEs (van Weering et al., 2012; Cullen and Steinberg, 2018; Weeratunga et al., 2020). SNX27 may direct these complexes to EEs by binding PI3P via its PX domain or by the coincidental recognition of cargo proteins via PDZ and FERM domains (Lauffer et al., 2010; Chandra et al., 2021). The PDZ domain binds transmembrane (TM) and cytosolic proteins with type I PDZ binding motifs (Gallon and Cullen, 2015), which is essential for the development of the cargo retrieval domain and also for the initiation of the curvature required for tubulation. Tubulation and retrieval of cargo into tubules are further promoted by the sequential recruitment of the ESCPE-1 complex, initiated by the binding of SNX1 or SNX2 to the F3-FERM subdomain (Yong et al., 2021; Simonetti et al., 2022). Apparently, Rab10 was recruited downstream of the platform created by SNX27:Retromer:ESCPE-1 complexes and facilitates tubule elongation, as indicated by recruitment of EGFP-SNX27-ΔF3 without recruitment of Rab10 in MCMV-infected cells (**Figure 3**).

An important function of the growing tubular membranes could be the concentration of viral envelope glycoproteins on membranes used for secondary envelopment (**Figure 6**). Both pM55 and pUL55, a major envelope glycoprotein gB of MCMV and HCMV, have a cytoplasmic short linear motif (SLiM) for association with the PDZ domain of SNX27 (**Supplementary Table S5**). In addition, gB of MCMV and HCMV, and gN (pM73) of MCMV have cytoplasmic SNX-BAR type II SLiM, while gM (UL100) of HCMV has type I SLiM for association with ESCPE-1 components (**Supplementary Table S5**). Thus, SNX27:Retromer:ESCPE-1 may enable retrieval of gB and gM/gN complexes but not retrieval of gH-based pentameric glycoprotein complex (Close et al., 2018) as gH has a very short cytoplasmic tail and other complex components are luminal. In addition to retrieval, prolonged tubulation associated with delayed membrane flux and inhibition of endosomal recycling (Tomaš et al., 2010; Karleuša et al., 2018; Zeltzer et al., 2018) may provide prolonged retention of viral envelope proteins within tubulating membranes. Retention of a tegument organizer could also provide the platform for the mobilization of tegument proteins to membranes and the accumulation of tegument in the form of a biomolecular condensate, which has been proposed as the driving force for envelopment of herpes simplex 1 (Metrick et al., 2020).

The persistent tubulation of endosomal recycling membranes within the inner AC may be essential for the envelopment of CMV capsids by the wrapping-based mechanism. This type of envelopment is suggested by EM studies (Maninger et al., 2011; Schauflinger et al., 2013; Taisne et al., 2019; Momtaz et al., 2021; Wedemann et al., 2022), but the mechanism to overcome physical constraints in such a complex model remained unclear. The growing tubular membranes of REs could wrap around large condensates of tegument material containing single or multiple capsids, similar to the development of phagophores. A recent study (Puri et al., 2023) shows that autophagosomes can originate from multiple foci of origin on REs that form finger- or octopus-like projections to wrap around the autophagosome substrate, followed by ESCRT-dependent closure required for further dynamin-mediated release of autophagosomes from this compartment. This mechanism is suitable for envelopment and can be utilized by CMVs, as evidenced by EM observations of wrapping-based envelopment and the requirements of ESCRT-III (Tandon et al., 2009; Streck et al., 2018) and dynamin (Hasan et al., 2018; Štimac et al., 2021) for CMV maturation and release of infectious virions. The analysis of the host cell signature within the virions (Mahmutefendić Lučin et al., 2022) provided in comprehensive proteomic (Birzer et al., 2020; Couté et al., 2020; Turner et al., 2020; Flomm et al., 2022) and lipidomic (Liu et al., 2011) studies on isolated virions indicate the use of membranes derived from REs.

Our study also shows that SNX27:Retromer:ESCPE-1-associated functions are required for CMV gene expression. SNX27 requirement was observed very early in infection and intensifies as the MCMV cycle progresses, eventually leading to a decrease in L gene expression and in release of infectious virions to a comparable magnitude. Therefore, it is likely that SNX27/Retromer/ESCPE-initiated tubulation within the pre-AC provides signaling platform that is essential for the surplus of MCMV gene products required for lytic infection (**Figure 6**). It is known that endosomes can serve as a signaling platform for internalized signaling receptors to drive many cellular processes, including receptors that utilize the CIE transport pathway (Murphy et al., 2009). SNX27:Retromer:ESCPE-1 complexes can regulate the cell surface level of many proteins and thereby control signaling processes (reviewed in Seaman, 2018). In addition, signal transduction from internal compartments can determine specific signaling consequences (Tsvetanova and von Zastrow, 2014; Willette et al., 2023). Thus, the retention of membrane flux associated with extensive tubulation can be exploited by CMVs not only to create structures for the secondary envelopment but also to regulate the progression of the replication cycle. Cargo retrieval mechanisms, including SNX27-dependent sorting, may play an active role in these processes, as SNX27 can recruit internalized signaling receptors via its PDZ and F3-FERM domains or recruit cytoplasmic signaling proteins via its PDZ, PX, and F3-FERM domains (Seaman, 2018).

In addition to the retention or recruitment of the host cell signaling machinery, the SNX27:Retromer:ESCPE-1-associated signaling platform can be established by retrieval of viral glycoproteins in tubular extensions. Several studies have shown that signaling from the PM occurs through gB-associated signaling axes, such as the recruitment of EGFR and β1- and β3-integrins (Chan et al., 2009; Campadelli-Fiume et al., 2016; Collins-McMillen et al., 2017; Yurochko, 2017; Fulkerson et al., 2020). These signaling events may persist from the intracellular compartment after their endocytic uptake. However, it is unlikely that the persistent signaling is related to the retrieval by the SNX27 PDZ domain, as the H112A mutation did not prevent L gene expression (**Figure 5**). Nevertheless, the endosome signaling may be prolonged by other mechanisms within the SNX27:Retromer:ESCPE-1 complex, including functions associated with the Retromer and the F3-FERM domain. As infection progresses, i.e. at 4-6 hpi, SNX27 dependence increases, coinciding with the timing of the onset of tubulation events associated with the establishment of pre-AC, and progresses during infection, as shown by the effects on pM57, viral DNA replication and L gene expression. These observations raise numerous new questions for further studies on CMV biology, particularly on the physiological role of the stepwise membrane reorganization known as the establishment of pre-AC and AC. The tremendous membrane reorganization and repositioning of membrane structures could create a new spatial order in the infected cell, as it is now known that the positioning of endosomes determines unique functional responses depending on the subcellular location and localization of signaling processes (Willette et al., 2023).

The effect of SNX27:Retromer:ESCPE complexes on Rab10 domain tubulation and viral gene expression suggests that these mechanisms play an important role in the progression of the CMV life cycle and possibly in envelopment. Other viruses also utilize SNX27:Retromer:ESCPE-1 to infect host cells or complete their assembly (Groppelli et al., 2014; Yin et al., 2016; Cardoso et al., 2020; Yang et al., 2022). However, there is very little data on their contribution to DNA viruses, mainly for the entry of human papillomaviruses (Bergant Marušič et al., 2012; Pim et al., 2015, 2021). An interaction between ppUL35, the tegument protein of HCMV, with SNX5, a component of ESCPE-1, has shown that endosomal retrieval contributes to HCMV replication (Maschkowitz et al., 2018). This study demonstrates that the inactivation of the SNX5-mediated retrograde route by binding ppUL35 is required for proper gB trafficking and virus replication. Since gB of MCMV and HCMV possess a SNX-BAR binding motif (**Supplementary Table S5**), the interaction of tegument with ESCPE-1 components is expected to be important for gB sorting. However, more detailed studies on the MCMV homolog (pM35) are required as very little is known about the cytoplasmic itinerary of pM35.

In conclusion, our study reveals an important function of SNX27:Retromer:ESCPE-1 complexes in the formation of a subset of tubular membranes within the AC of a CMV-infected cell. Existing knowledge of these complexes suggests that their function may be important in the processes of secondary envelopment and egress of CMV viruses. Notably, our study reveals a novel and important additional function of membrane tubulation within the AC in controlling the viral replication program within the infected cell. Although the adaptation of cellular signaling pathways in CMV-infected cells has been the subject of many studies, very little is known about signaling from membranous structures within the AC. Therefore, further studies are needed to elucidate these processes. Understanding them could open the window for further development of host-directed antiviral therapy. Namely, many antiviral drugs which target viral components fail due to the rapid generation of drug-resistant viruses (Kumar et al., 2020). An alternative to this could be targeting host cell factors that control viral replication. In this context, signaling pathways are of particular importance as numerous drugs have been developed and many of them are currently in clinical trials. In addition, the signaling capacity of cell types can be studied to explain differences in the ability to develop a productive infection, cellular heterogeneity in the response to infection (Suomalainen and Greber, 2021), and establishment and reactivation from latency.

## Data Availability

The original contributions presented in the study are included in the article/**Supplementary Material**. Further inquiries can be directed to the corresponding authors.
