# Supplementary material for: SNX27:Retromer:ESCPE-1-mediated early endosomal tubulation impacts cytomegalovirus replication
Source: Front Cell Infect Microbiol. 2024 Sep 18;14:1399761. doi: 10.3389/fcimb.2024.1399761 (PMC11445146; doi:10.3389/fcimb.2024.1399761)
Supplement: Supplementary file 1 [file SupplementaryFile1.pdf]

Supplementary Material

1.1 Supplementary Figures.

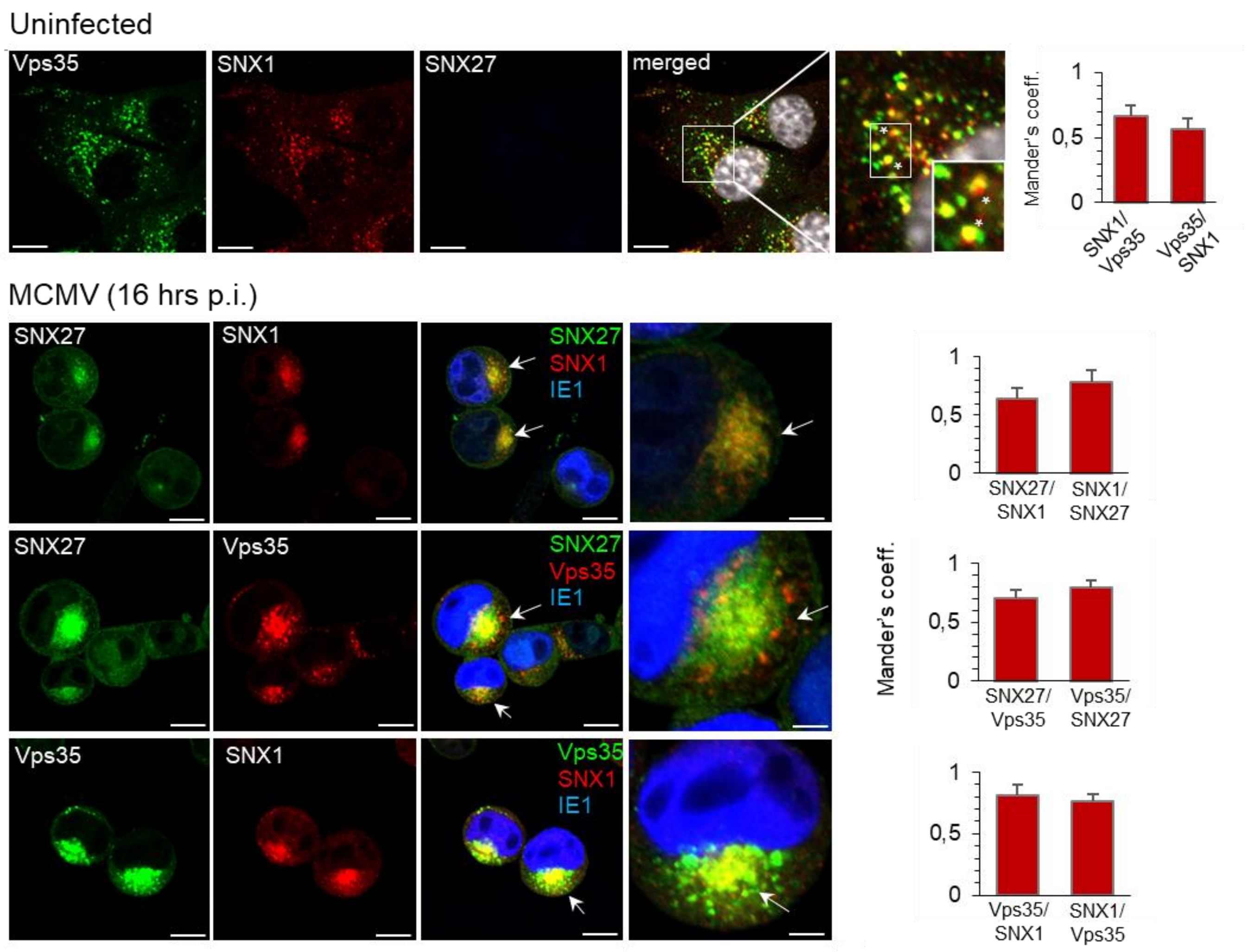

Figure S1. Colocalization analysis of endogenous SNX27, Vps35 and SNX1 (*related to Figure 1*). Uninfected cells were triple stained for SNX27, Vps35 and SNX1 (DAPI-stained nucleus is shown in gray), whereas MCMV-infected (Δm138-MCMV, MOI of 10, 16 hpi) cells were stained for a combination of host cell markers and additionally for pIE1 expression to indicate infection. Mander's coefficients (M1 and M2) were calculated on the entire image stacks to quantify colocalization. The data represents the mean ± SD of at least 10 cells from two independent experiments. Arrows indicate pericentriolar pre-AC. Asterisk indicates Vps35/SNX1 partitioning in the same compartment. Bars, 10 µm (lower magnification) and 5 µm (higher magnification).

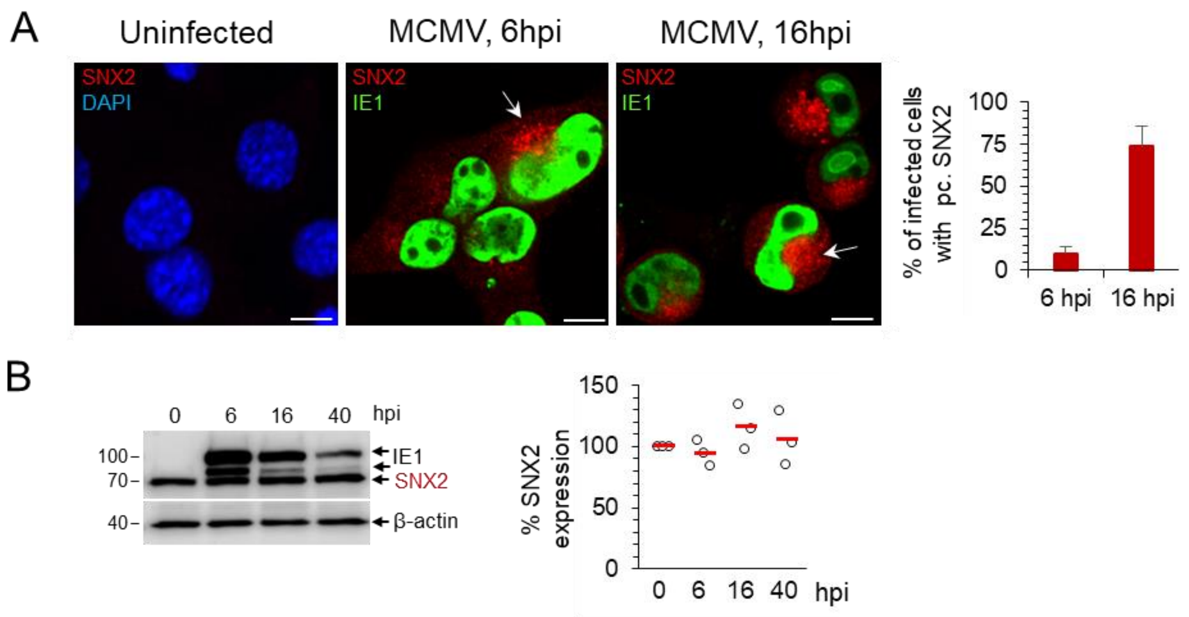

Figure S2. SNX2 expression in MCMV-infected cells (*related to Figure 1*). (**A**) Balb3T3 fibroblasts were infected with Δm138-MCMV (MOI of 10) or left uninfected. Samples were then fixed, permeabilized, and labelled with anti-SNX2 antibody (red), anti-IE1 antibody (green), or DAPI (blue) and analyzed by confocal microscopy (left panel). Bars, 10 µm. The percentage of MCMV-infected cells with pericentriolar SNX2 accumulation was quantified and presented as the mean ± SD (right panel). (**B**) Kinetics of SNX2 expression in MCMV-infected cells as determined by Western blot analysis. Shown are the individual results (empty circles) and the average (red bars) from three independent experiments. The mean values are shown as red bars.

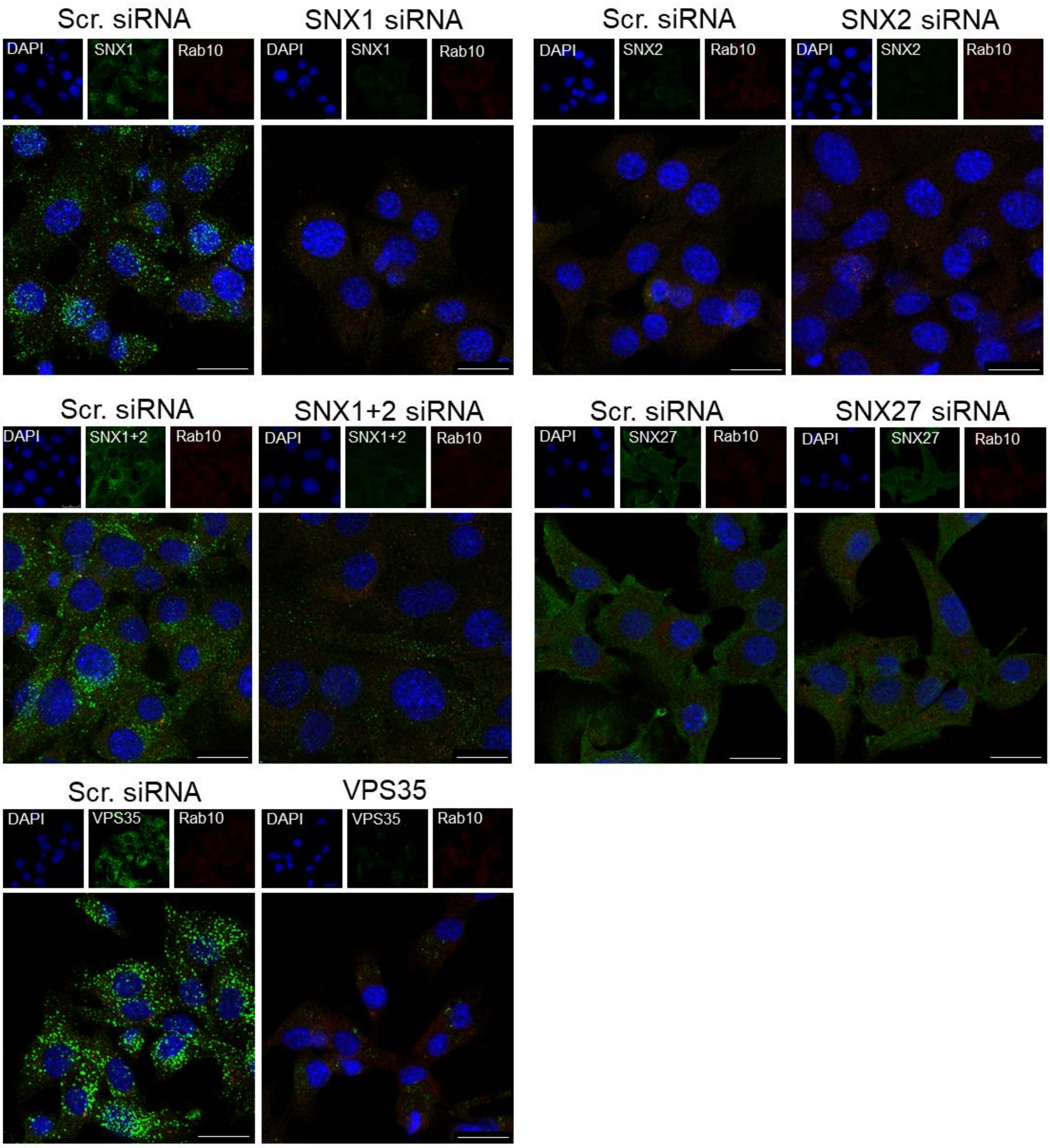

Figure S3. The siRNA depletion does not enhance Rab10 visualization in uninfected Balb3T3 cells (*related to Figure 2*). Balb3T3 fibroblasts were transfected with scr-siRNA or siRNA for SNX27, Vps35, SNX1, and SNX1+2. After 48 hours, cells were fixed with PFA, permeabilized, and labeled with appropriate antibodies against SNX1, SNX2, SNX27, Vps35 and SNX1+2 (green) combined with antibodies against Rab10 (red). Cells were then washed, incubated with AF-conjugated secondary antibodies, mounted, and analyzed by confocal microscopy. DAPI was used to label the nucleus. Bars, 10 μm.

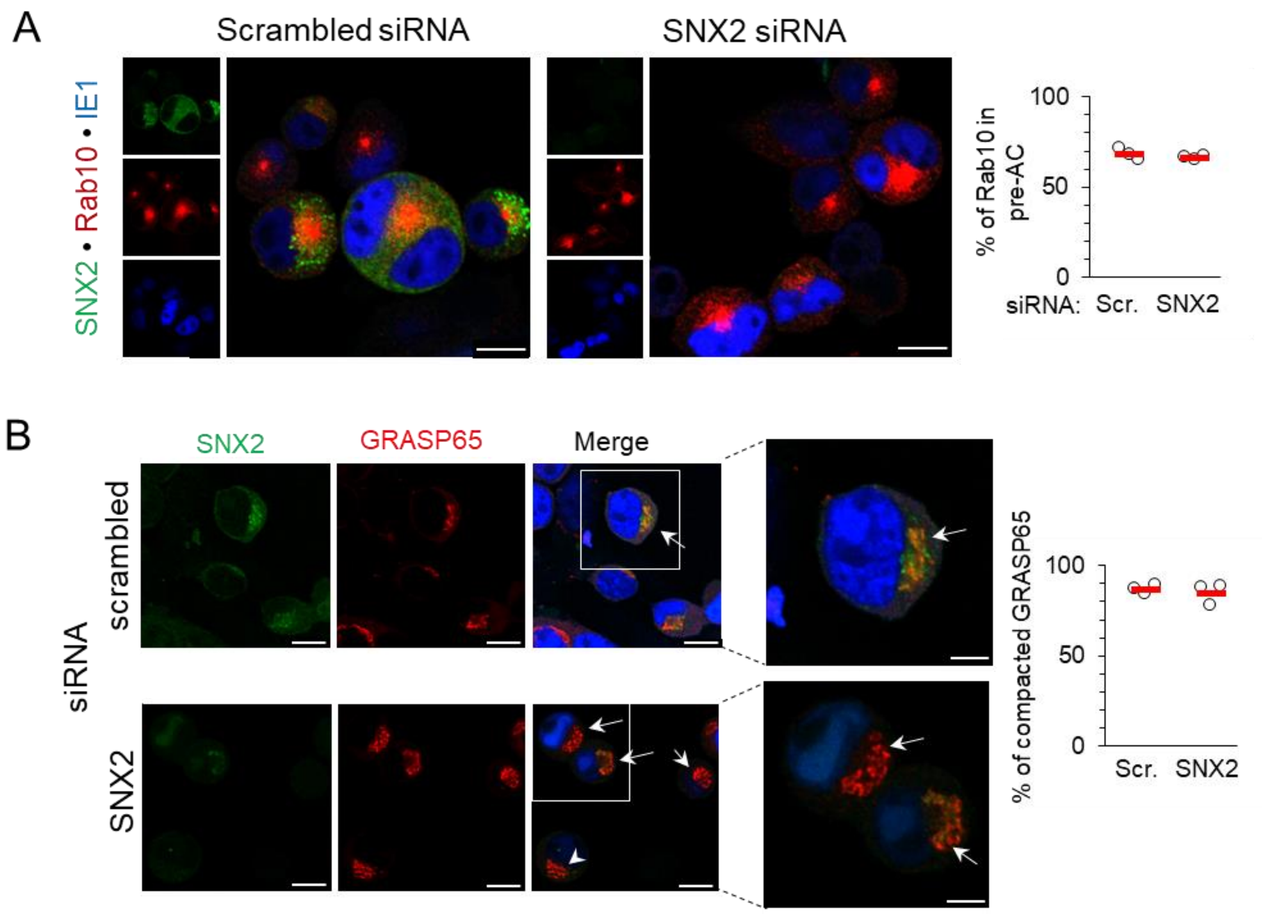

Figure S4. Effect of SNX2 siRNA depletion on pericentriolar accumulation of Rab10 and GRASP65 in MCMV-infected cells (*related to Figure 2*). After transfection with siRNA (48 h) and MCMV infection (Δm138-MCMV, MOI 10, 16 hpi), Balb3T3 cells were fixed, permeabilized, and labeled with anti-SNX2 (green), anti-Rab10 (**A**) or GRASP65 (**B**) (red) and anti-IE1 (blue) antibodies. After visualization with the non-cross-reactive appropriate AF-conjugated secondary antibodies, the samples were analyzed by confocal microscopy. The percentage of cells with accumulated pericentriolar Rab10 or GRASP65 from three independent experiments is shown in the graphs as empty circles. The mean values are shown as red bars. Arrows indicate GRASP65 in the ring-like formation representing pre-AC. Bars, 5 (higher magnification, **B**) and 10 μm.

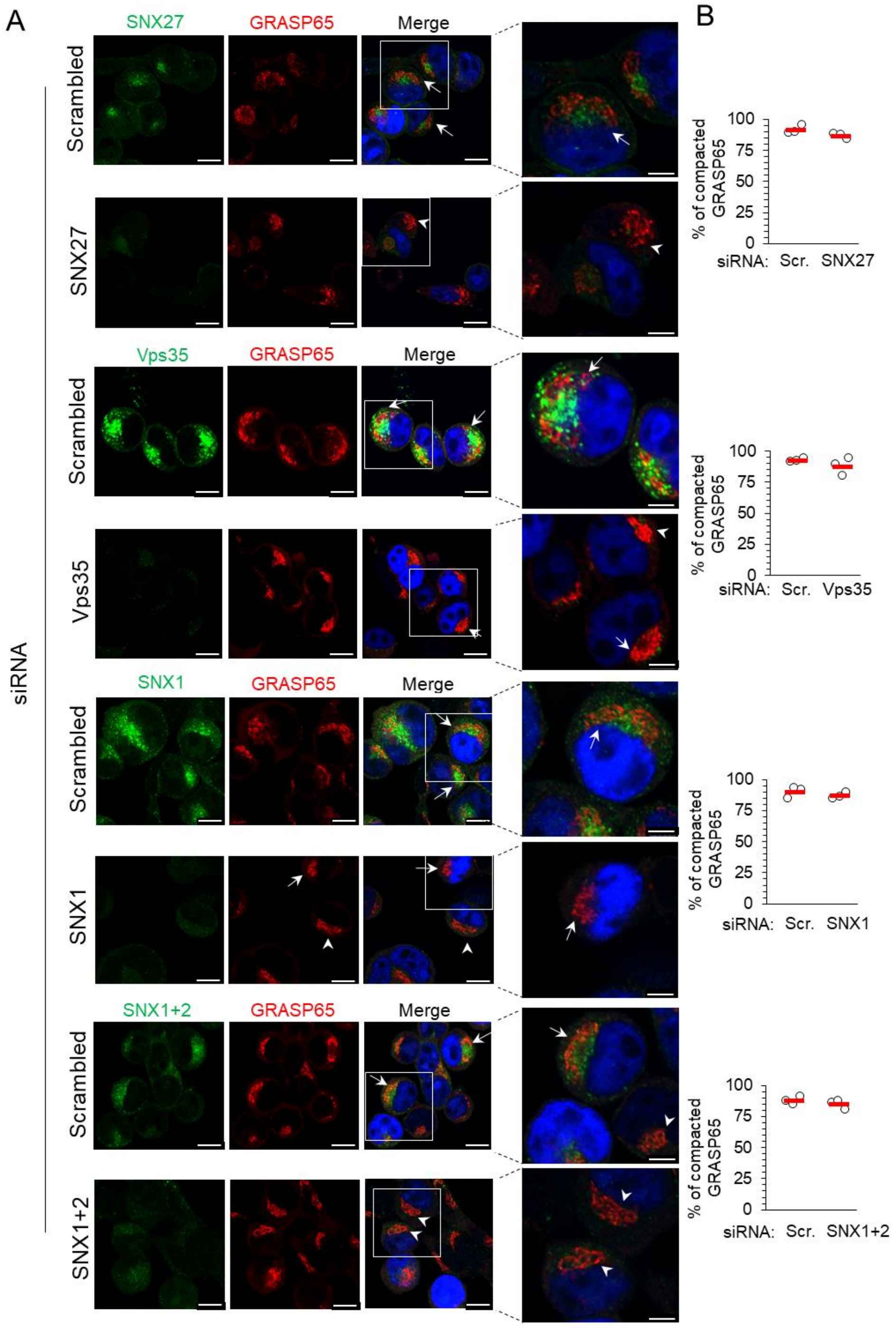

Figure S5. siRNA depletion of SNX27, Vps35, SNX1 and SNX1+2 has no effect on Golgi reorganization in the early phase of MCMV infection. (**A**) Balb3T3 fibroblasts were transfected with scr-siRNA or with siRNA for SNX27, Vps35, SNX1, and SNX1+2. After 48 hours, cells were infected with Δm138-MCMV (MOI of 10) and fixed for 16 hpi with PFA, permeabilized and labelled with the corresponding antibodies against SNX27, Vps35, SNX1, or SNX1+2 (green), combined with anti-GRASP65 (red) and anti-IE1 (blue). After staining with corresponding AF-conjugated, non-cross-reactive secondary antibodies, the samples were mounted and analyzed by confocal microscopy. Part of the compacted GRASP65 showed a ring-like formation representing pre-AC (arrows), while others did not (arrowheads). Bars, 5 and 10 µm. (**B**) The percentage of cells that developed compacted Golgi from three independent experiments is shown as empty circles in the graphs. The mean values are shown as red bars.

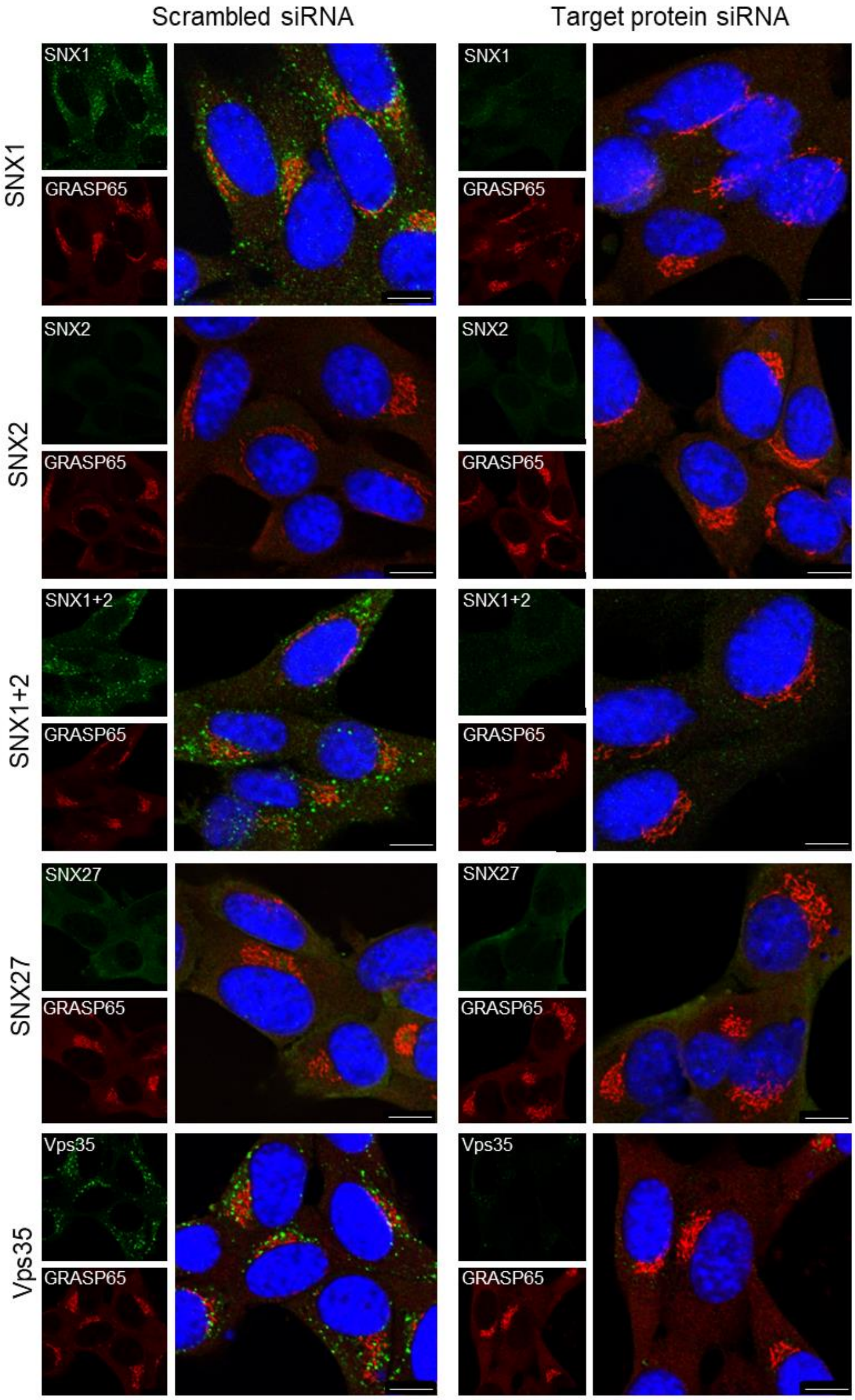

Figure S6. siRNA depletion of SNX1, SNX1+2, SNX27, and Vps35 has no effect on the Golgi in uninfected cells (*related to Figure 2*). Balb3T3 fibroblasts were transfected with scr-siRNA or with siRNA for SNX1, SNX2, SNX1+2, SNX27, and Vps35. After 48 h, the cells were fixed with PFA, permeabilized and stained with the corresponding antibodies against SNX1, SNX2, SNX1+2, SNX27, and Vps35 (green) in combination with anti-GRASP65 (red). After staining with appropriate AF-conjugated, non-cross-reactive secondary antibodies, the samples were mounted and analyzed by confocal microscopy. DAPI was used to label the nucleus. Bars, 10 μm.

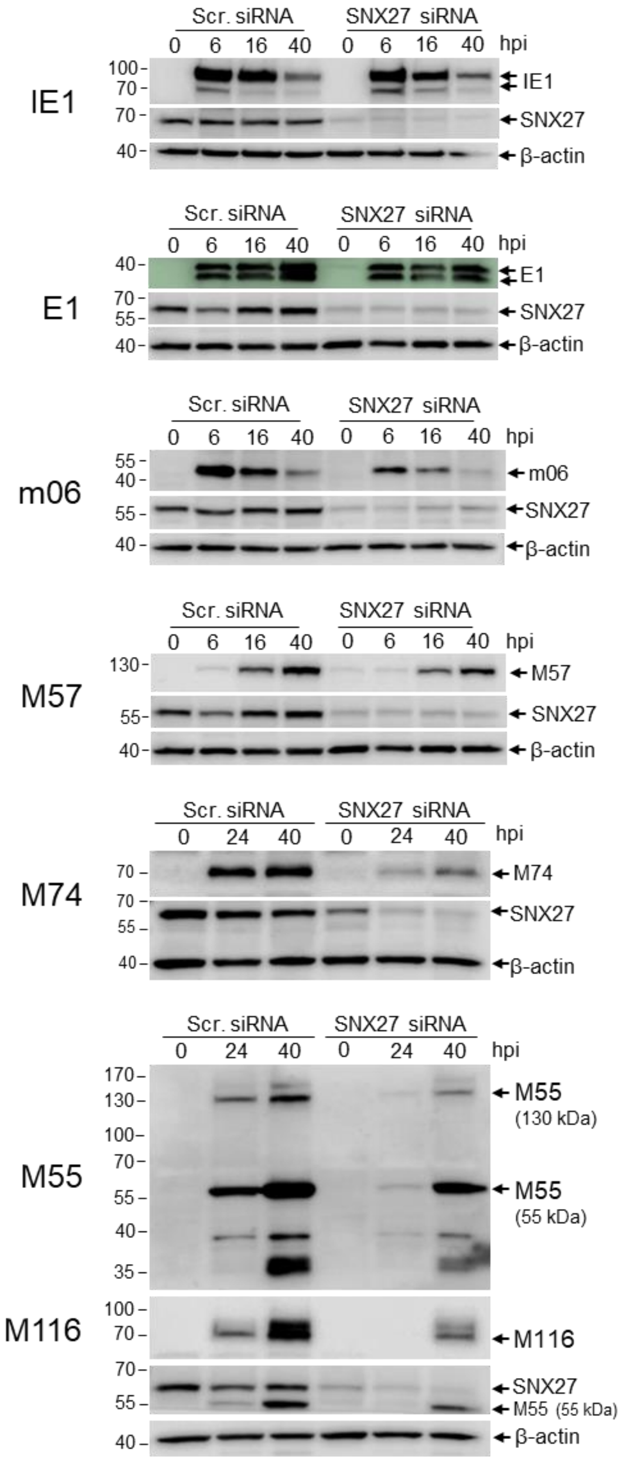

Figure S7. Depletion of SNX27 inhibits progression through MCMV replication cycle (*related to Figures 4C and 4E*). Balb3T3 fibroblasts were transfected with scrambled or SNX27 siRNAs. After 48 hours, cells were infected with Δm138-MCMV (MOI of 10) and analyzed by Western blot at 0, 6, 16 and 40 hpi. Shown are images of representative Western blots of pIE1, pE1, pm06, pM57, pM74, pM55, and pM116 together with SNX27 and β-actin on the same membrane. The blot of pM116 was developed sequentially after pM55. The position of molecular weight markers was labeled on the left.

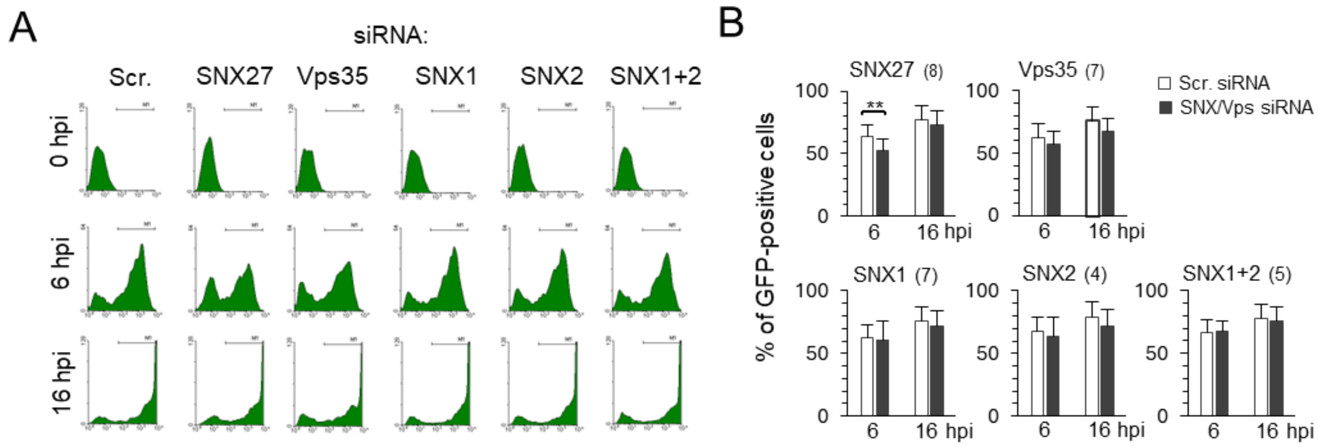

Figure S8. Depletion of SNX27 moderately impairs the progression through the early phases of infection (*Related to Figure 4B*). Balb3T3 fibroblasts were transfected with scrambled-, SNX27-, Vps35-, SNX1-, SNX2- and SNX1+2-siRNA and infected with the recombinant virus expressing GFP (C3X-GFP-MCMV; MOI of 10) after 48 hours. GFP expression was determined by flow cytometry at 6 and 16 hpi. Results are presented as flow cytometric profiles of a representative experiment (**A**) and as a percentage of GFP-positive cells (**B**), showing the mean±SD of four to eight independent experiments (the number of experiments is indicated in parentheses). Statistical significance was determined using a two-tailed paired Student t-test (**$p < 0.01$; *$p < 0.05$).

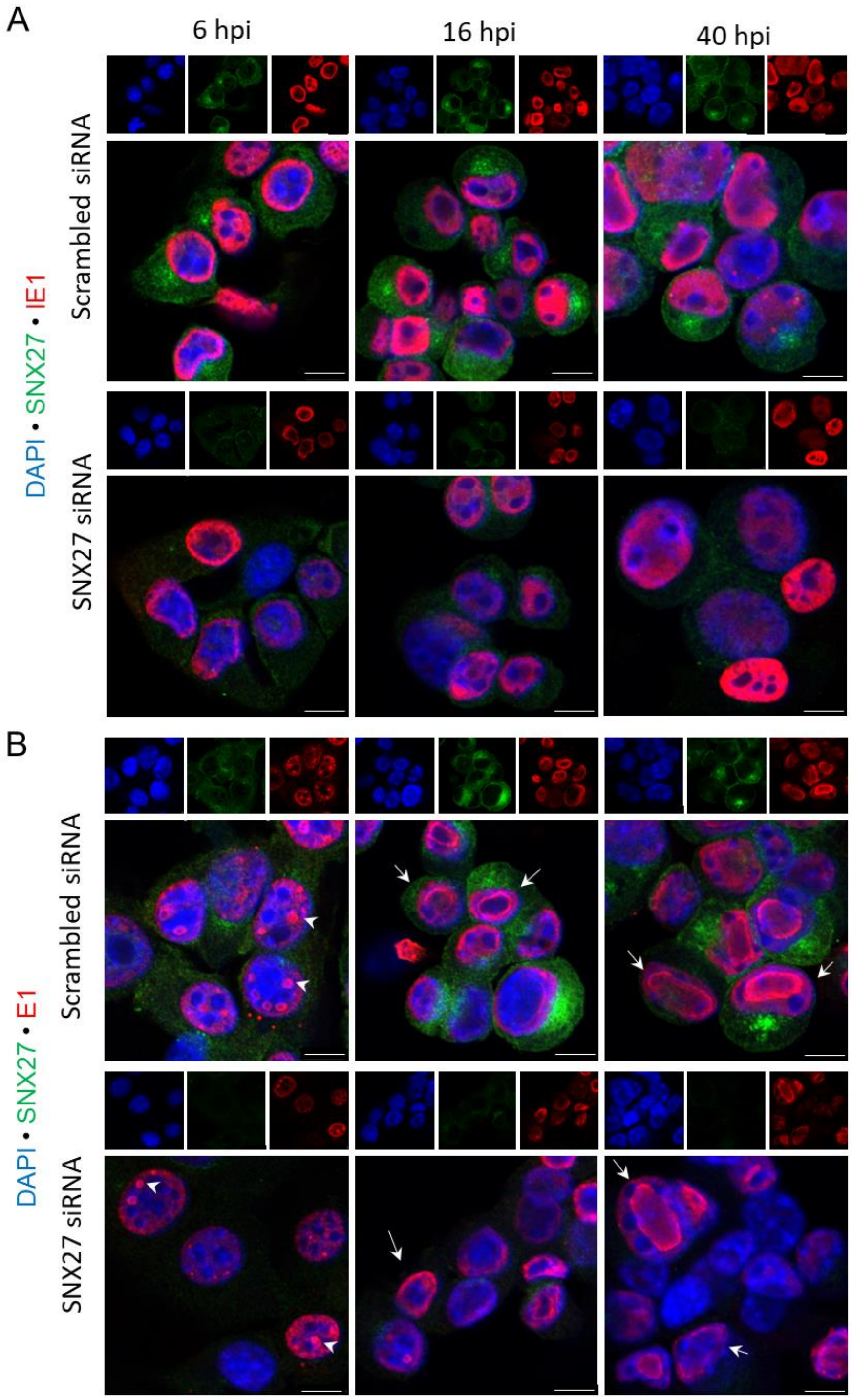

Figure S9. Immunofluorescence analysis of pIE1 and pE1 expression in scr-siRNA- and SNX27 siRNA-depleted cells (*related to Figure 4B*). **(A-B)** Balb3T3 fibroblasts were transfected with scrambled or SNX27 siRNAs. After 48 hours, the cells were infected with Δm138-MCMV (MOI of 10) and analyzed by immunofluorescence for the expression of pIE1 **(A)** and pE1 **(B)**. At 6, 16 and 40 hpi, cells were stained with DAPI (blue) and antibodies against SNX27 (green) and pIE1 or pE1 (red), followed by isotype-specific, non-cross-reactive secondary antibodies. Shown are representative images taken with confocal microscopy through the focal plane. Arrows indicate large hollow pE1 patterns, and arrowheads represent punctate nuclear structures and spherical nuclear assemblies **(B)**. Bars, 10 μm.

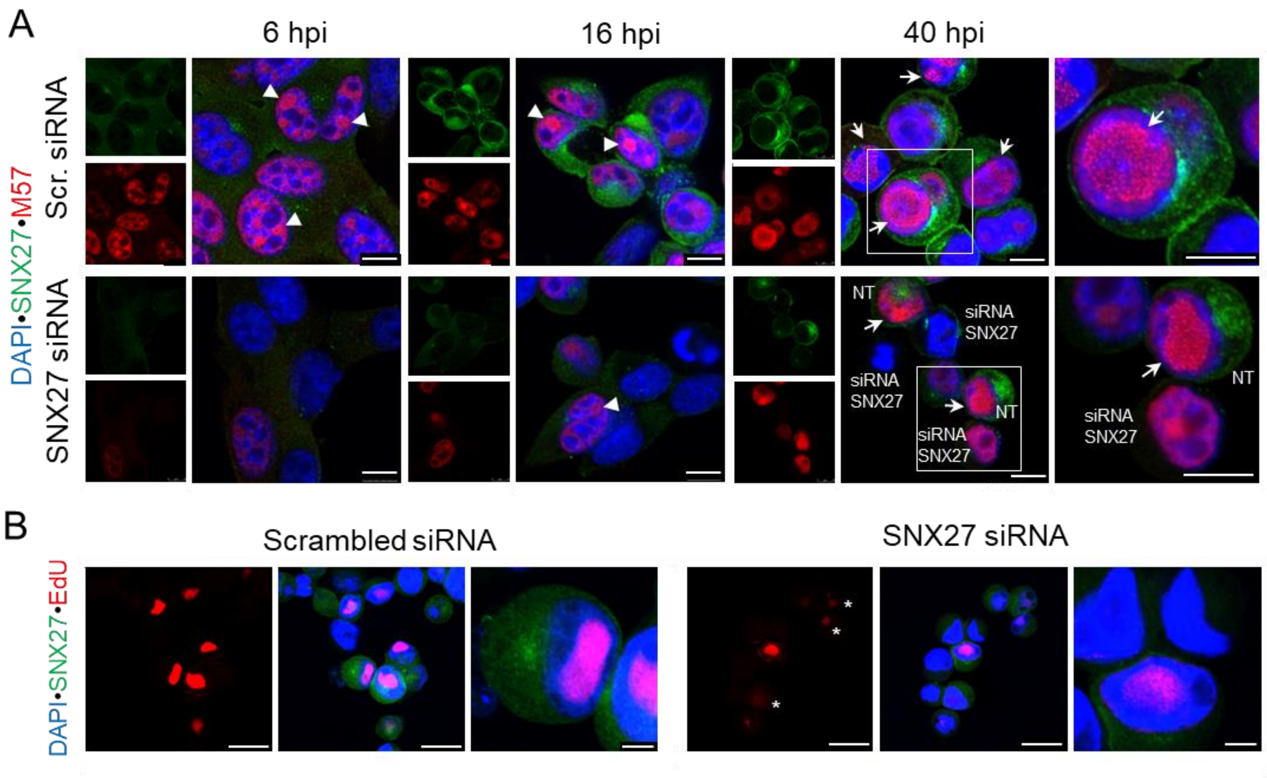

Figure S10. Depletion of SNX27 inhibits progression through the E phase of infection (*related to Figure 4B*). Balb3T3 fibroblasts were transfected with scrambled or SNX27 siRNAs. (**A**) After 48 hours, cells were infected with Δm138-MCMV (MOI of 10) and analyzed by immunofluorescence at 6, 16 and 40 hpi. After labeling with anti-SNX27 (green) and anti-M57 antibodies (red), cells were stained with isotype-specific, non-cross-reactive fluorochrome-conjugated secondary antibodies and analyzed by confocal imaging. DAPI was used to label the cell nuclei. Shown are the representative confocal images. The arrowheads indicate the early phase, and the arrows indicate the mature biomolecular condensates of pM57 in the nucleus. (**B**) After 48 hours, cells were infected with Δm138-MCMV and labeled 16-24 hpi with 10 µM EdU, followed by staining with antibodies against SNX27 and pIE1, and visualization of EdU-labeled DNA with the click reaction. Asterisks indicate siRNA SNX27-treated cells with low EdU labeling. NT, Nontransfected. Bars, 10 µm.

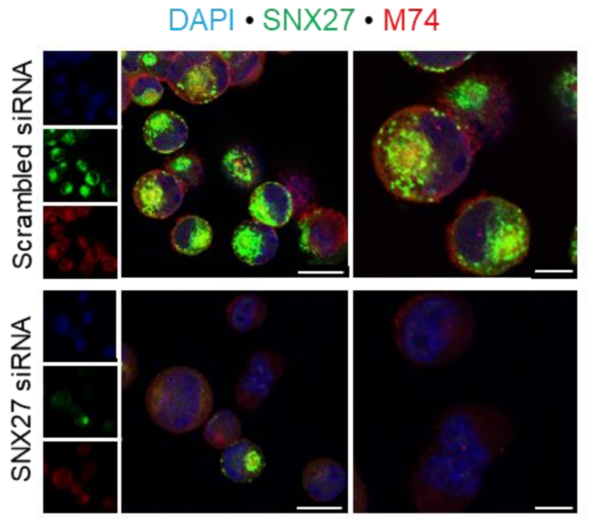

Figure S11. Depletion of SNX27 reduces the expression of late MCMV proteins (*related to Figure 4E*). Representative immunofluorescence images of pM74 (red), SNX27 (green) and DAPI (blue) in scr-siRNA-treated and SNX27-depleted Balb3T3 cells at 40 hpi, imaged by confocal microscopy through the focal plane. Bars, 10 µm.

1.2 Supplementary tables

Table S1. Quantification# of SNX1, SNX27 and VPS35 colocalization in uninfected and MCMV-infected (16 hpi) cells (*related to the data shown in Figures 1 and S1*).

	**SNX1 with Vps35**	**Vps35 with SNX1**	**SNX27 with SNX1**	**SNX1 with SNX27**	**SNX27 with Vps35**	**Vps35 with SNX27**
Uninfected (Balb3T3)	0.66 ± 0.07	0.57 ± 0.06	ND	ND	ND	ND
MCMV-infected (Balb3T3)	0.76 ± 0.05	0.81 ± 0.08	0.64 ± 0.09	0.78 ± 0.09	0.7 ± 0.07	0.79 ± 0.06
Uninfected (NIH3T3 EGFP mSNX27)	ND	ND	0.69 ± 0.08	0.74 ± 0.1	0.79 ± 0.05	0.78 ± 0.11
MCMV-infected (NIH3T3 EGFP mSNX27)	ND	ND	0.64 ± 0.12	0.61 ± 0.16	0.74 ± 0.09	0.75 ± 0.13

#Mander's coefficients were presented as mean ± standard deviation (SD) as described in Material and Methods, section 2.5. ND – no data

Table S2. Cell viability after treatment with SNX1, SNX2, SNX1+2, SNX27 and Vps35 siRNAs[#] (*related to the data shown in Figure S8).*

siRNA	0 hpi	6 hpi	16 hpi
Scrambled	95.5±3.2%	90.1±4.8%	84.2±6.2%
SNX1	95.1±2,1%	90,3±3.1%	82.4±5.7%
SNX2	94.8±3.8%	89.9±6.7%	89.1±4.9%
SNX1+2	94.8±2.3%	90.2±1.2%	88.7±4.8%
SNX27	93.6±2.6%	91.6±3.3%	94.5±3.8%
Vps35	95.8±1.3%	90±4.9%	91.3±6.3%

[#]Balb3T3 fibroblasts were transfected with scrambled siRNA or siRNA for SNX1, SNX2, SNX1+2, SNX27, and Vps35. After 48 hours, the cells were infected with C3X GFP MCMV (MOI of 10) and cell viability was determined at 0, 6 and 16 hpi. Propidium iodide-negative cells were classified as viable, and the results are presented as mean±SD of three independent experiments.

Table S3. Immunofluorescence analysis of MCMV protein expression in control and SNX27-depleted cells# (*related to the data shown in Figure 4B*).

MCMV protein:		pIE1		pE1		pM57		pM74	
siRNA:		Control	SNX27	Control	SNX27	Control	SNX27	Control	SNX27
6 hpi	mean±SD#	91.2 ± 5.3	81.07 ± 4.01	86.98 ± 12.3	76.45 ± 13.01	44.08 ± 4.68	20.5 ± 2.22	ND	
	p	*0.05071**		*0.3663*		*0.00426***			
16 hpi	mean±SD	90,6 ± 2.77	82.77 ± 3.15	90.53 ± 4.84	86.14 ± 7.45	70.47 ± 9.01	41.3 ± 5.27	ND	
	p	*0.0321**		*0.455*		*0.01082***			
40 hpi	mean±SD	87.41 ± 8.81	86.68 ± 6.01	91.3 ± 8.78	78.18 ± 11.9	71.95 ± 9.92	44.75 ± 8.58	49.05 ± 14.8	29.07 ± 5.84
	p	*0.4861*		*0.2001*		*0.0229**		*0.0212**	

#Results are presented as mean ± SD of three independent experiments. Statistical significance was determined using a two-tailed paired Student t-test (**p* < 0.05; ***p* < 0.01; *p**** < 0.001). ND – No data.

Table S4. The expression of MCMV proteins in SNX27-depleted cells[a] (*related to the data shown in Figures 4C and 4E*).

		pIE1 (6)[b]	pE1 (6)	pM57 (4)	pm06 (4)	pM74 (4)	pM55 (4)		pm116 (4)
							55 kDa	130 kDa	
		siRNA SNX27 / siRNA ctrl.							
6 hpi	Mean ± SD	0.79 ± 0.13	0.87 ± 0.05	ND	0.34 ± 0.24	ND	ND	ND	ND
	p	*0.005*	*0.059*		*0.0134*				
16/24 hpi[c]	Mean ± SD	0.78 ± 0.18	0.74 ± 0.11	0.43 ± 0.18	0.35 ± 0.04	0.29 ± 0.15	0.12 ± 0.09	0.037 ± 0.04	0.065 ± 0.08
	p	*0.0229*	*0.023*	*0.008*	4.6×10^{-6}	*0.0029*	2.9×10^{-4}	2.8×10^{-5}	*0.00013*
40 hpi	Mean ± SD	0.62 ± 0.11	0.8 ± 0.1	0.50±0.16	*0.28* ± 0.06	0.34 ± 0.09	0.033 ± 0.14	0.27 ± 0.12	0.46 ± 0.04
	p	*0.006*	*0.015*	*0.009*	1.78×10^{-5}	*0.00076*	*0.0025*	*0.0013*	*0.00034*

Balb3T3 fibroblasts were transfected with scrambled siRNA or SNX27 siRNA and infected with Δ138 MCMV (10 MOI) after 48 hours. The expression of MCMV proteins was detected by Western blot. The results are shown in the respective kinetics (means±SD) in the form of fold changes compared to the control siRNA. Statistical significance was determined using a two-tailed paired Student t-test (***$p < 0.001$; **$p < 0.01$; *$p < 0.05$). ND, no data.

[a] The value for control cells (scrambled siRNA) was calculated as 1).
[b] The number of independent experiments is given in brackets.
[c] 16 hpi for pIE1, pE1, pM57 and pm06; 24 hpi for pM74, pM55 and pm116 MCMV proteins.

Table S5. Assumed SLiMs in MCMV and HCMV transmembrane glycoproteins.[a]

**MCMV envelope proteins**			
	**SNX protein**	**Canonical SLiM sequence**	**SLiM in cytoplasmic tail of CMV protein**
**M55 (gB)**	SNX27-PDZ	[ED][ST]X[AILMFVPG] (Cullen & Steinberg, 2018)	ESGP, DTSG, DSDF
	SNX-BAR	[FYW]X[FY]X_{3-15}ΦXΦ (Yong et al., 2020)	FPYATQTAVQYAPP
**M73 (gN)**	SNX-BAR	[FYW]X[FY]X_{3-15}ΦXΦ (Yong et al., 2020)	WCYKAFTSDTAKG
**HCMV[b] envelope proteins**			
	**SNX protein**	**Canonical SLiM sequence**	**SLiM in cytoplasmic tail of CMV protein**
**UL55 (gB)**	SNX27-PDZ	[ED][ST]X[AILMFVPG] (Cullen & Steinberg, 2018) [ST][ST]X[AILMFVPG] (Cullen & Steinberg, 2018)	DTSL, SSDA, STAA
	SNX-BAR	[FYW]X[FY]X_{3-15}ΦXΦ (Yong et al., 2020)	FPYATQTAVQYAPP
**US100 (gM)**	SNX-BAR	[ILMV]X[FY]X[RK]X_{2-13}ΦXΦ (Yong et al., 2020)	VRYFRGRGSG

[a]Protein sequences were extracted from UniProt (https://www.uniprot.org/), transmembrane domains were found using DeepTMHMM. To search the SNX-BAR binding motifs (SBMs) in human proteome, we used TMHMM 2.0 (https://services.healthtech.dtu.dk/services/TMHMM-2.0/; accessed on March 9, 2024) (Krogh et al., 2001), and predict transmembrane proteins: (Yong et al., 2020): As described in (Yong et al., 2020), two different patterns were searched for transmembrane proteins, mainly envelope proteins, of CMV. For single-pass TM proteins, the cytoplasmic tail was searched, while for multipass TM proteins only the last cytoplasmic region was checked.

[b] strain AD169.

Supplementary References:

Cullen, P. J., & Steinberg, F. (2018). To degrade or not to degrade: mechanisms and significance of endocytic recycling. *Nature Reviews Molecular Cell Biology*, *19*(11), 679–696. https://doi.org/10.1038/S41580-018-0053-7

Krogh, A., Larsson, B., Von Heijne, G., & Sonnhammer, E. L. L. (2001). Predicting transmembrane protein topology with a hidden Markov model: application to complete genomes. *Journal of Molecular Biology*, *305*(3), 567–580. https://doi.org/10.1006/JMBI.2000.4315

Yong, X., Zhao, L., Deng, W., SunID, H., Zhou, X., Mao, L., Hu, W., Shen, X., Sun, Q., Billadeau, D. D., XueID, Y., & JiaID, D. (2020). *Mechanism of cargo recognition by retromer-linked SNX-BAR proteins*. https://doi.org/10.1371/journal.pbio.3000631
